# VOCs Adsorption and Exchange Properties in Bispidine‐Based Mn(II) 1D CPs Made of Orthogonally Oriented Linear Chains

**DOI:** 10.1002/chem.71316

**Published:** 2026-07-07

**Authors:** Meriem Goudjil, Martina Lippi, Sara Camorali, Chiara Pelosi, Alessandro Sayed, Luca Bernazzani, Patrizia Rossi, Paola Paoli, Massimo Cametti

**Affiliations:** ^1^ Department of Industrial Engineering University of Firenze Firenze Italy; ^2^ Department of Chemistry, Materials and Chemical Engineering “Giulio Natta” Politecnico di Milano Milano Italy; ^3^ Department of Chemistry and Industrial Chemistry University of Pisa Pisa Italy; ^4^ Institute For the Chemistry of Organometallic Compounds‐ICCOM Italian National Research Council‐CNR Pisa Italy

**Keywords:** 1D coordination polymers, adsorption, bispidine ligands, SC‐to‐SC transformations, volatile organic compounds (VOCs), X‐ray diffraction

## Abstract

The combination of bispidine ligand **L1** and Mn(II) produces CP 1‐MeCN with a one‐dimensional (1D) architecture unprecedented for bispidine‐based CPs. Single‐crystal X‐ray diffraction (SC‐XRD) reveals a robust framework of orthogonally oriented ribbon‐like chains in the tetragonal space group *P*4_2_2_1_2 with solvent‐filled perpendicularly intersecting channels that account for about 30% of the unit‐cell volume. Volatile organic compound (VOC) exchange on SC proceeds via clean SC‐to‐SC transformations with nitrobenzene (NB), chlorobenzene (ClBz), and 1,2‐dichlorobenzene (DCB), but not with toluene (Tol). The corresponding microcrystalline powder, **1‐MeCN^Pwd^**, and its thermally generated evacuated amorphous phase, **1‐amorph^Pwd^**, were tested as well. In particular, **1‐amorph^Pwd^** adsorbs ca. 1.6–1.9 mmol/g of aromatic NB, ClBz, and DCB (but only 0.7 for Tol) and up to 2.5 mmol/g of MeCN, under saturated vapor conditions at r.t. Although these uptake capacities are comparable to those of previously reported bispidine‐based CP sorbents, the preservation of the orthogonal chain packing motif upon guest uptake and the distinct interaction mechanism with the tested VOCs highlight a different role of chain topology and packing in governing sorption and offer new design criteria for the development of novel VOC‐capturing materials.

## Graphical abstract

Combining the chiral bispidine ligand **L1** with MnCl_2_ affords coordination polymers (CPs) with an unusual topology for this family of compounds, consisting of orthogonally packed ribbon‐like one‐dimensional (1D) chains. Their behavior was investigated through single‐crystal‐to‐single‐crystal exchange with aromatic volatile organic compounds (VOCs) and vapor adsorption by the evacuated amorphous phase, showing how this particular packing of arrays governs guest uptake and selectivity.

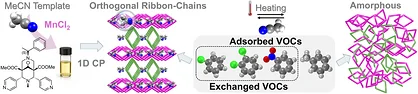

## 1 Introduction

Volatile organic compounds (VOCs) are pervasive atmospheric and indoor pollutants linked to acute and chronic health effects and to secondary processes such as ground‐level ozone formation and photochemical smog [1, 2, 3]. Their sources span petrochemical refining, chemical and pharmaceutical manufacturing, vehicle emissions, and everyday indoor materials and products, making their mitigation a continuing challenge. Conventional abatement strategies (thermal/catalytic oxidation, biofiltration) can be effective but often at the expense of energy efficiency, selectivity, or operational simplicity, prompting sustained interest in adsorptive capture as a complementary approach. Porous adsorbents, including zeolites, activated carbons, silica gels, and polymeric resins, remain useful benchmarks, yet their selectivity, regenerability, and tunability can be limited [4, 5]. Coordination polymers (CPs) and, in particular, metal–organic frameworks (MOFs) introduced a novel paradigm in which crystallinity, high surface area, and chemical programmability can be harnessed for targeted adsorption [6, 7]. Numerous MOFs display impressive VOC uptakes aided by open metal sites, hydrogen‐bonding, and *π*–*π* interactions with aromatic linkers and, more recently, halogen‐bond donors engineered into the pore environment [8]. However, these uptake capacities are typically established on thermally activated materials under controlled, anhydrous conditions, and several challenges remain to transfer their performance to ambient, competitive, and humid environments. At the same time, the notion that permanent high porosity is a prerequisite for optimal adsorption, especially at low VOC partial pressure, can be challenged. Hence, the need to investigate novel classes of dynamic solids that do not meet all the standard requirements, which are believed to be mandatory for efficient adsorption, but that at the same time could undergo guest‐induced reorganization, “making space” for the incoming adsorbate without the presence of pre‐existing large and accessible channels [9, 10, 11, 12, 13]. One‐dimensional (1D) CPs are especially intriguing in this context. Early reviews by Chen and Suslick [14], and later by Leong and Vittal [15], highlighted how simple metal–ligand building blocks can assemble into chains, ladders, ribbons, or helices whose supramolecular packing gives rise to novel properties and guest‐induced flexibility. These studies demonstrated that 1D CPs can combine crystallinity, modular synthesis, and dynamic solid‐state responses, placing them conceptually between rigid, usually 3D, MOFs and more labile supramolecular or molecular solids. Indeed, 1D CPs present robust, coordinatively bonded chains assembled through weak, noncovalent interchain interactions, thereby forming 3D supramolecular networks capable of exhibiting dynamic behavior in response to external stimuli. This type of architecture often yields a relatively flat energy landscape relative to the packing, with multiple close minima accessible upon specific stimuli and under mild conditions. Basically, this feature may enable reversible phase transformations upon exchange or adsorption of several types of guests to which the material is exposed. Takamizawa and coworkers were key pioneers in the field, demonstrating that 1D CPs can operate as efficient gas‐adsorbing host systems. In their seminal study on the benzoate–pyrazine chain 1D arrays, they showed that CO_2_ uptake generates distinct inclusion phases and reversible phase transitions, with the CO_2_ positions and binding sites directly located by single‐crystal x‐ray diffraction (SC‐XRD) analysis [16, 17]. This concept was subsequently extended to a series of related 1D CP hosts that were shown to be capable of capturing several different types of guests such as NO_x_, SO_2_, and alcohols [18, 19, 20]. Taken together, this body of work established 1D CPs as dynamic, gas and VOC adsorbents in which adsorption, phase behavior, and host–guest interactions can be studied and correlated at atomic resolution. Later on, Noro and co‐workers exemplified selective gas adsorption in flexible 1D Cu(II) CP systems, in which polar Cu‐PF_6_ units act as specific binding sites for CO_2_ and C_2_H_2_; crystallographic studies and sorption isotherms revealed that subtle changes in anion coordination and axial ligation allow fine‐tuning of adsorption properties [21]. More recently, a flexible 1D Cu(II) carboxylate–pyrimidine polymer has been used to demonstrate how co‐adsorbed gases may regulate water vapor uptake kinetics, with CO_2_ slowing H_2_O adsorption far more than N_2_ and thereby highlighting the interplay between host flexibility, sorption kinetics, and multicomponent gas environments [22]. The adsorption applications by 1D CPs for gas, vapors, and larger molecular guests, also extracted from solution, have been recently reviewed, providing a comprehensive view on these early and modern examples of 1D CP adsorbers [23].

Bispidine‐based CPs have recently been proposed as promising novel VOC adsorbers [23, 24]. Bispidine compounds, featuring the rigid, highly preorganized 3,7‐diazabicyclo[3.3.1]nonane scaffold, provide a particularly powerful platform to obtain a series of highly functionalized ligands. Indeed, they offer the possibility of extensive functionalization, allowing a precise tuning of donor set and denticity, steric and electronic factors, which govern metal–ligand interactions. Several examples of variably functionalized bispidine ligands employed to obtain molecular complexes have been reported by Comba et al., and others [25, 26, 27]. More recently, suitably functionalized bispidines have been demonstrated to be able to form CPs when reacting with several different metal ions [28, 29]. From a structural point of view, bispidine‐based CPs with Mn(II), Hg(II), and Cu(II) have shown, so far, diverse topologies, going from ribbon‐like to zig‐zag, helical, and rare polycatenane arrays [24]. In all such cases, SC‐XRD characterization determined parallelly aligned arrays weakly interacting with each other via weak hydrogen bonding (HB) (e.g., CH_arom_···O═C/COOCH_3_, COOCH_3_···O─C). From a functional point of view, ribbon‐like and polycatenane motifs were frequently found to be highly responsive in terms of solvent exchange and adsorption under ambient conditions. In contrast, zig‐zag arrays tended to be comparatively more inert, highlighting that topology is a crucial factor [30]. Quantitatively, aromatic and aliphatic VOC uptakes for these 1D systems typically reached ca. 1–3 mmol/g, figures that, while being lower than those found for the best‐in‐class MOFs at saturation (> 15 mmol/g), were achieved under ambient exposure, with no prior activation and evacuation, in the presence of humidity and atmospheric gases. Against this backdrop, uncovering new structures and topologies is both scientifically and technologically relevant, and expanding beyond known motifs may provide routes to enhanced adsorption properties.

Here, we report that the combination of ligand **L1** (Scheme 1) with MnCl_2_ salt leads to the formation of a Mn(II) bispidine‐based CP that features an unprecedented 1D topology consisting of orthogonally oriented 1D arrays and which demonstrates pronounced solvent adsorption and exchange under ambient conditions. We elucidated its structure by SC‐XRD, analyzed its packing architecture in the context of previously reported bispidine CPs, and directly compared it with analogous Mn(II) CPs made with parallelly oriented arrays. Moreover, we tracked its guest uptake and exchange properties by SC‐to‐SC transformations, and on powder phases via P‐XRD and TGA‐FTIR/MS techniques.

**SCHEME 1:**
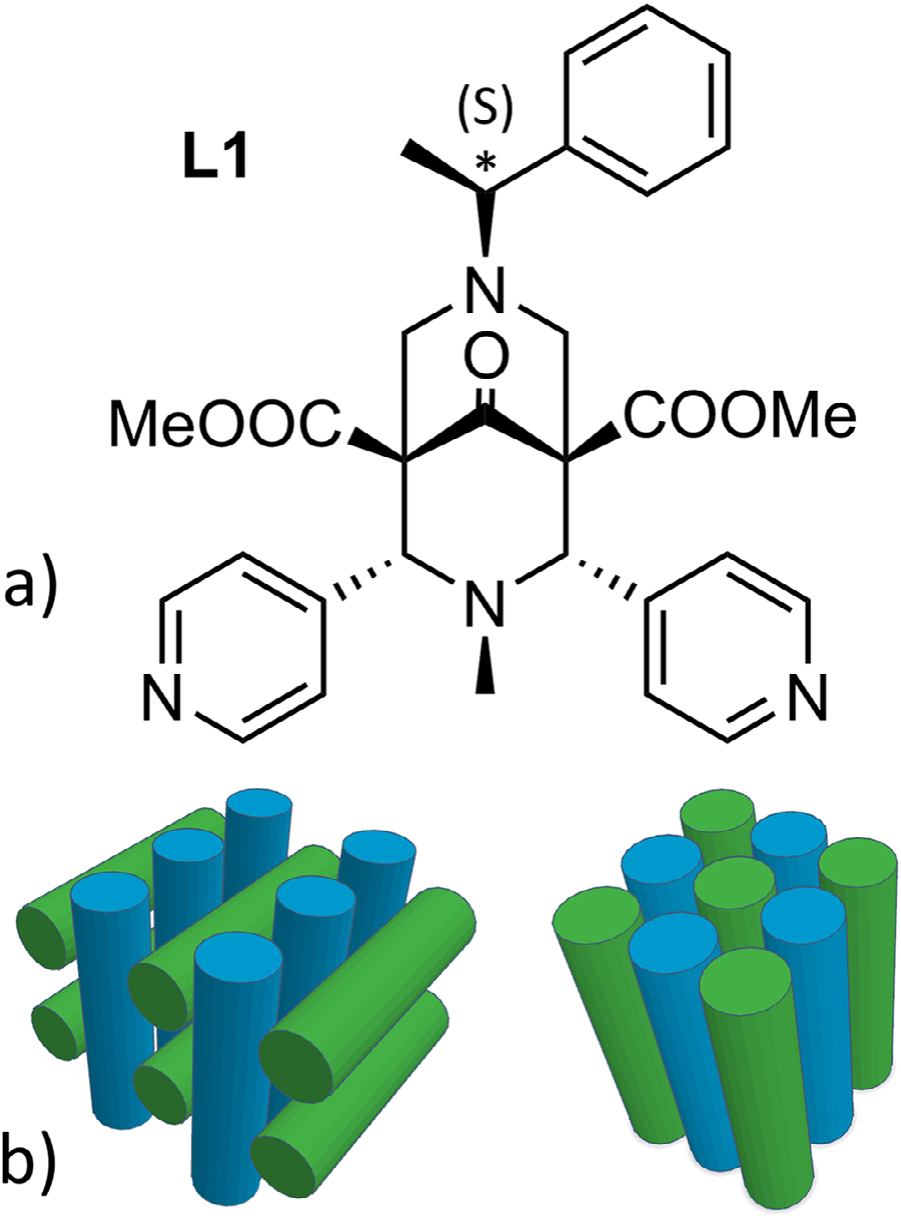
(a) Molecular formula of ligand **L1**. (b) Cartoon depicting the different relative orientation of the 1D arrays in this work compared to previously reported 1D CPs.

## 2 Results and Discussion

### 2.1 SC‐XRD Characterization of L1 and 1‐MeCN

Good‐quality SCs of **L1** were obtained within 24 h via slow diffusion; the ligand (20 mg) dissolved in 2 mL of EtOH was layered on top with 1 mL of DEE. Details of its structure and crystal packing are provided in the Supporting Information (Tables S1 and S2, and Figures S1–S3). Slow evaporation of a solution of **L1** and MnCl_2_ in MeCN:MeOH (7:1) solvent mixture produced good quality SCs of a novel 1D CP named as **1‐MeCN^SC^** (Figure 1), which revealed an unprecedented topology compared to previous bispidine‐based CPs. This CP crystallizes in the tetragonal crystal system, adopting the *P*4_2_2_1_2 space group (Table S3), the highest symmetry reported so far for bispidine‐based CPs. Each bispidine ligand **L1** bridges two Mn(II) centers through its pyridinic sidearms (Figure 1a). The pyridinic nitrogen atoms coordinate Mn(II) ions in the equatorial plane defined by four symmetry‐related N donors generated by 2‐fold axes parallel to the [110] and [1‐10] directions. Symmetry‐equivalent chloride ions occupy the axial sites along the [001] direction, so that each Mn(II) center adopts a distorted octahedral {MnN_4_Cl_2_} coordination environment, comprising four equatorial pyridine N atoms from four ligands and two apical chloride ions (Figure 1a). Overall, the ligand‐to‐metal stoichiometry is found to be 2:1 ratio generating a CP of composition {[Mn(**L1**)_2_Cl_2_]·4(MeCN)}_∞_, which comprises four co‐crystallized MeCN solvent molecules (Table S3) entrapped within the voids generated by the assembly of 1D ribbon‐like arrays (Figure 1b). Although the 1D dimensionality and the presence of linear ribbon‐like assemblies are quite usual a recurrence in bispidine‐based CPs, especially when Mn(II) metal is used, on this occasion, the relative arrangement of the 1D chains markedly differs from the previously reported structures (Figure S4). The 1D ribbon chains are not all parallel to each other, and indeed extend along the (110) and (−110) planes as shown in Figure 1c. Chains parallel to (110) propagate via lattice translation along the *b*‐axis, whereas those parallel to (−110) extend along the *a*‐axis. This bidirectional growth results in an overall packing characterized by orthogonal ribbon‐like arrays, which are interrelated by a 4_2_‐screw axis parallel to [001] (Figure S5). Among the arrays, a distinct arrangement in which ester groups interact with aromatic moieties rather than with one another can be identified (vide infra). The orthogonal 1D ribbon chains lying in the (−110) planes are linked to those in the (110) planes, along the *c*‐axis direction, through weak O···H contacts involving the terminal oxygen atoms of the ester groups (O2 and O5) and the pyridine CHs (Table S5 and Figure 2a). In all the studied CPs, including those deriving from solvent‐exchange experiments, **1‐NB^SC^**, **1‐ClBz^SC^**, and **1‐DCB^SC^** (vide infra), interchain O···H interactions are weak and comparable in terms of distance and angular geometry (Tables S5–S8). Additionally, the orthogonal chains interconnect via weak C─H···Cl contacts observed between the methyl‐hydrogen of the ester groups and the Mn‐bound chlorine atoms (Table S5 and Figure 2a). These contacts exhibit slight elongation upon solvent exchange (Tables S5–S8), suggesting a degree of structural flexibility in response to guest molecules (vide infra). This packing architecture is significantly different from previously reported bispidine‐based CPs, not only in the overall topology, but also in terms of underlying inter‐array interactions. Whereas interchain packing in bispidine CPs is typically dominated by O···H contacts involving ester groups, these interactions are considerably weaker in the present case. The orthogonal arrangement of 1D chains, combined with weak interchain interactions, creates well‐defined interstitial voids that impart distinctive functional properties to the framework, particularly for solvent exchange. Upon virtual removal of guest solvent molecules, all the CP structures reported in this work consistently reveal a network of perpendicularly oriented 1D wavy channels that trace the openings defined by the ribbon‐like topology. These voids exhibit a cross‐section of approximately 7.5 Å and occupy nearly 30% of the total unit‐cell volume (Figure 1d), a value that represents the median of the virtual voids distribution in these CPs and in other Mn(II) bispidine‐based CPs (CCDC Refcodes: RIMZUL [9], LABQEO, and LABQUE [11]). The MeCN molecules confined within the ribbon space form weak hydrogen bonds, including one C–H···Cl contact between the MeCN‐methyl group and Cl2 (Table S5 and Figure 2b), along with a C─H···N contact between the MeCN‐nitrogen atom toward the methyl (C24A–H24C···N1A) hydrogen atom of the framework (Figure 2b and Table S5). The short H···N distance of 2.630(4) Å and near‐linear angle of 156(1)° for C24C–H24A···N1A indicate a relatively more pronounced directional character compared to the C─H···Cl solvent‐chain interaction.

**FIGURE 1:**
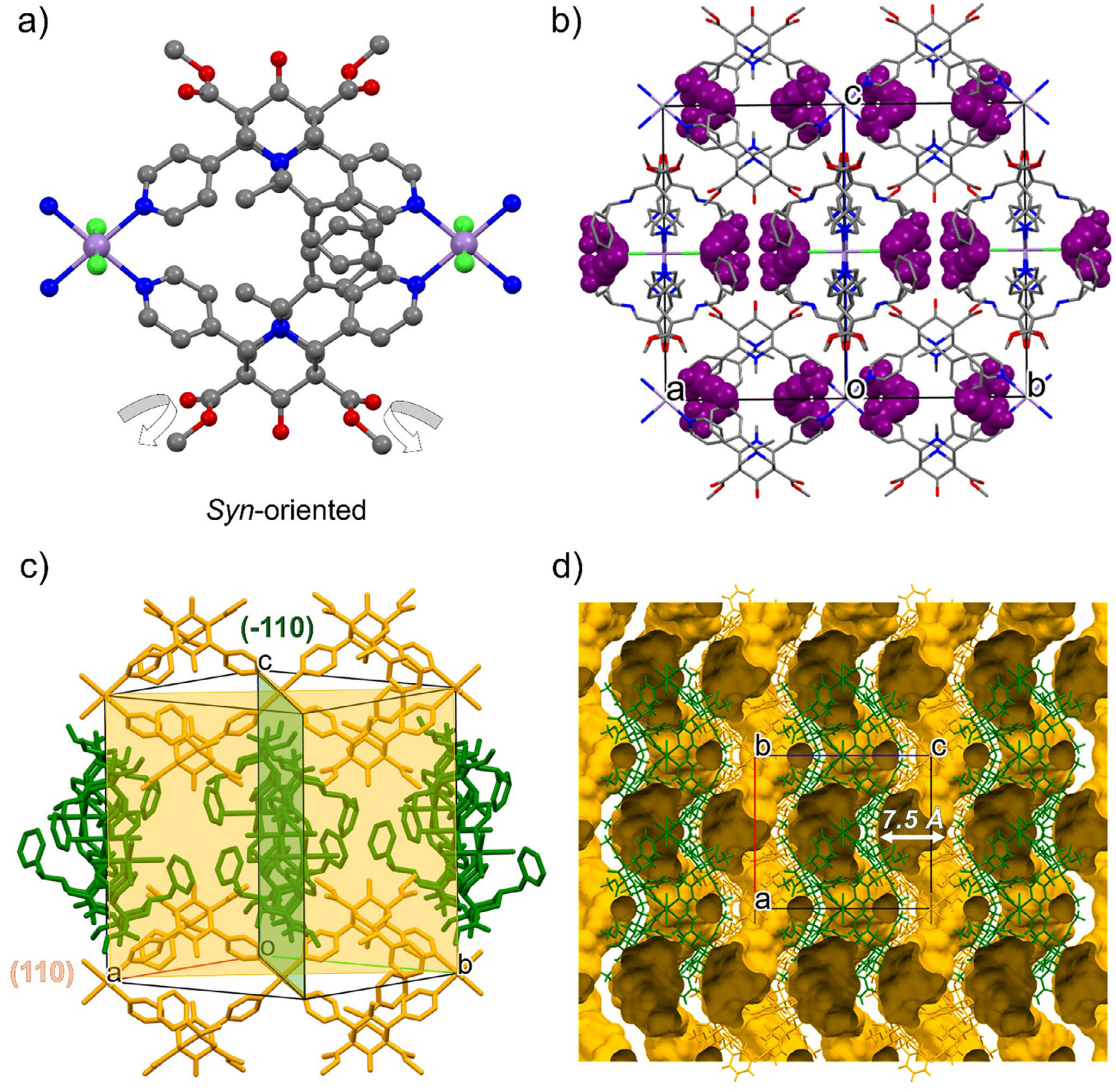
View of the SC‐XRD structure of **1‐MeCN^SC^**: (a) Ball and stick representation of the ribbon‐like motif, ester groups are *syn*‐oriented within the bispidine core. Color code: C = gray; N = blue; O = red; H = white; Cl = green; Mn(II) = purple. (b) Location of MeCN molecules within the CP framework. (c) Perspective view highlighting the orthogonal orientation of the 1D ribbon chains, and (d) virtual voids extending as wavy channels (cross section ca. 7.5 Å). H atoms are omitted for clarity in parts (a–c).

**FIGURE 2:**
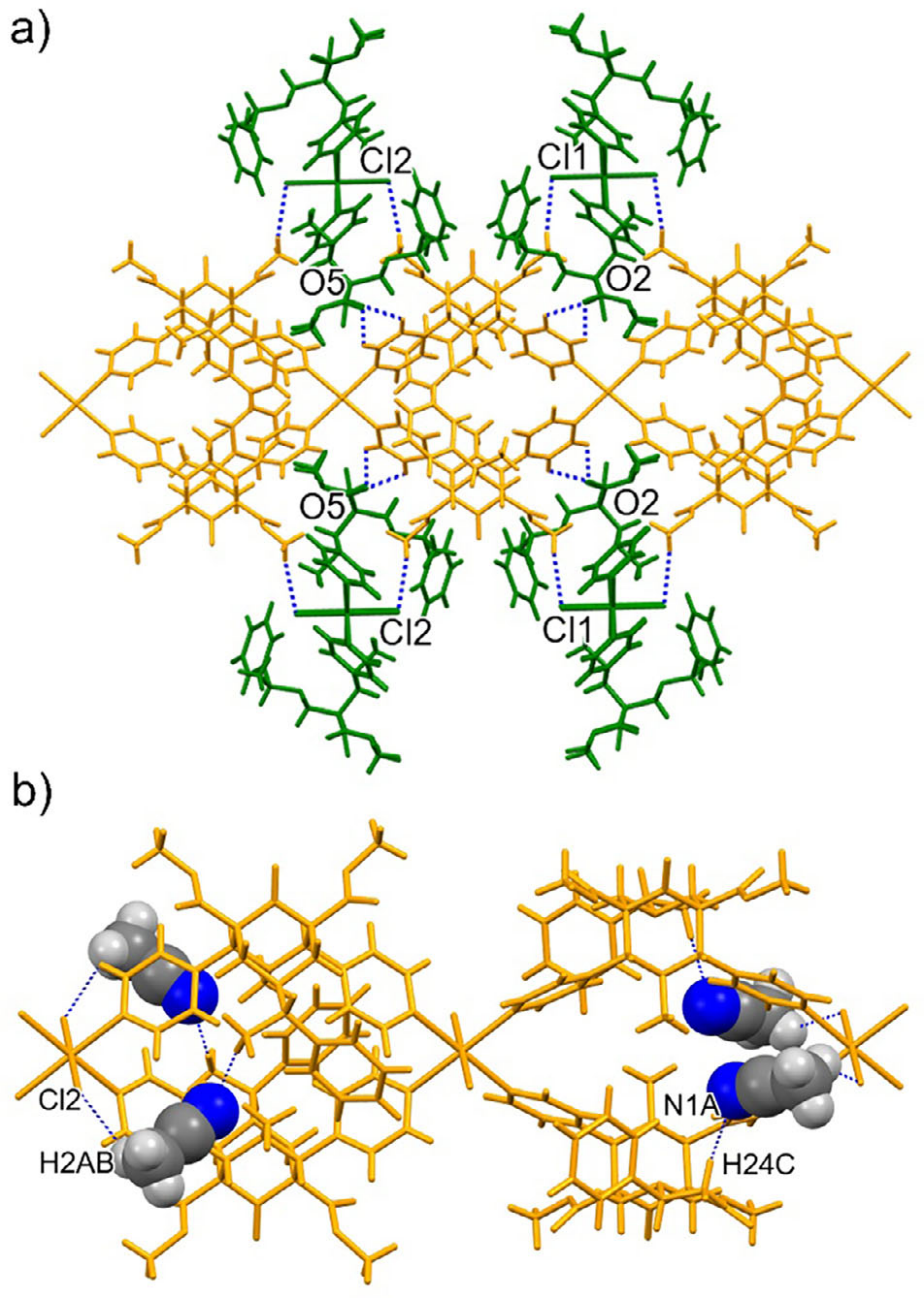
Packing views in **1‐MeCN^SC^** showing the intermolecular contacts (dashed lines): (a) interchain contacts involving C─H···O and C─H···Cl hydrogen bonds that link the orthogonal 1D ribbon chains along the *c*‐axis, (b) solvent–framework interactions, highlighting C─H···N and C─H···Cl contacts between MeCN molecules and the CP ribbon space.

### 2.2 Exposure of **1‐MeCN^SC^** to VOCs: SC‐to‐SC transformations

SCs of **1‐MeCN^SC^** were then tested by exposure to saturated atmospheres of selected VOCs (nitrobenzene = NB; chlorobenzene = ClBz; 1,2‐dichlorobenzene = DCB, and toluene = Tol) in order to evaluate the possibility of SC‐to‐SC transformations upon VOCs exchange. Carefully selected and characterized SCs of **1‐MeCN^SC^** were then examined to confirm structural integrity and high crystalline quality, and then exposed to vapors within a closed chamber. SC‐XRD analysis of the materials after 14 days showed that, in the cases of NB, ClBz, and DCB, the exchange transformations were successful. In particular, the initially included four MeCN molecules were replaced by 2, 1.4, and 0.66 molecules per formula unit of NB, ClBz, and DCB, respectively. Thus, novel SC species **1‐ClBz^SC^**, **1‐DCB^SC^**, and **1‐NB^SC^** were obtained. In the case of the latter, the amount of adsorbed solvent was estimated from the residual electron density observed in the Fourier maps and was treated using the solvent‐mask procedure; further details are provided in the Supporting Information. In contrast, SC‐XRD data indicated that Tol has not been adsorbed under the same conditions. Based on this initial assessment, this behavior may be related to the steric or electronic factors, which could affect a solvent's ability to adopt orientations favorable for host–guest stabilization [31]; however, based solely on these data, different solvent‐dependent kinetics of the process cannot be ruled out. The adsorbed ClBz and DCB molecules occupy spatial regions analogous to those of MeCN in **1‐MeCN^SC^**, see Figures 1b and S6, thereby enabling the formation of weak C─H···Cl contacts with the 1D chains. Nevertheless, their interaction patterns within the polymeric framework exhibit subtle variations due to the disorder and steric profile of ClBz compared to DCB. It is worth noting that in **1‐ClBz^SC^**, guest molecules are predominantly disordered over two distinct sites, designated as 1S and 3S, with site occupancy factors (s.o.f.) of 0.30 each. At site 1S, ClBz molecules function exclusively as H‐bond donors, engaging with chlorine atoms Cl1 and Cl2 situated between adjacent 1D chains aligned parallel to the (−110) planes. These interactions are mediated via C4SA–H4SA···Cl1 and C2SA–H2SA∙∙∙Cl2 contacts (Table S7), effectively linking neighboring chains along the [−110] direction, see Figure 3a. At site 3S, ClBz molecules adopt a distinct orientation within the framework, as illustrated in Figure 3b. Their interaction with the 1D chains is mediated through weak C─H···π contacts, specifically between the aromatic hydrogen H5SC of ClBz and the centroid of the phenyl ring of the (S)‐(−)‐α‐methylbenzylamino moiety (C25A–C30A), with a contact distance of 3.191(3) Å and an angle of 119(1)° (Figure 3b). In **1‐DCB^SC^**, the incorporated DCB molecules (s.o.f. = 0.33) adopt a distinct bridge mode along the [−110] direction. They simultaneously engage in a donor‐type H‐bond with Cl2 (C5S–H5S···Cl2) on one side, and an acceptor‐type interaction with a neighboring chain via C29A–H29A···Cl2S on the opposite side (Figure 3c and Table S8), thereby establishing a unique dual‐mode connectivity between the 1D ribbon chains. As to the general structural overview of these CPs, the geometric parameters observed in all **1‐MeCN^SC^**, **1‐NB^SC^**, **1‐ClBz^SC^**, and **1‐DCB^SC^** (Mn─Cl, Mn─N, and intra‐ribbon Mn···Mn distance, etc.) are comparable and consistent with previously reported bispidine‐based Mn–CPs (Table S4) [9, 10, 11]. Importantly, at variance with previously reported bispidine‐based 1D CPs, comparison among the P‐XRD patterns of **1‐MeCN^SC^**, **1‐ClBz^SC^**, **1‐DCB^SC^**, and **1‐NB^SC^** simulated from the SC‐XRD data (Figure S9) shows that the solvent identity has a negligible effect on the packing of the 1D ribbon‐like chains.

**FIGURE 3:**
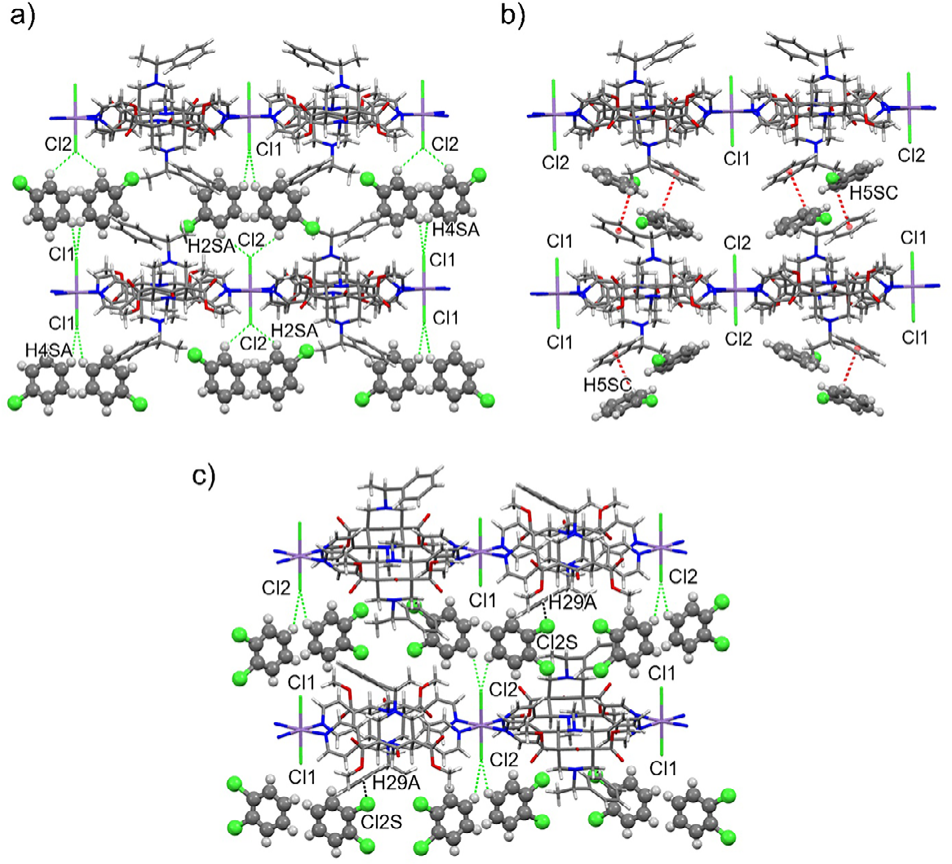
Visualization of solvent‐mediated C─H···Cl interactions within the 1D ribbon chains. (a) In **1‐ClBz^SC^**, ClBz molecules labeled as “1S” (s.o.f. = 0.30) act solely as H‐bond donors. (b) Those labeled “3S” (s.o.f. = 0.30) form only C─H∙∙∙π stacking with the 1D chains of the CP. (c) In **1‐DCB^SC^**, the DCB molecules (s.o.f. = 0.33) adopt a dual‐interaction mode, simultaneously engaging in one donor and one acceptor H‐bond.

### 2.3 Synthesis and P‐XRD Characterization of **1‐MeCN^Pwd^**

The CP in the form of microcrystalline powder, **1‐MeCN^Pwd^**, was obtained by fast synthesis methods, that is, by adding a **L1** solution in MeCN to a concentrated solution of Mn(II) in MeOH at r.t. The mixture was left under stirring for 12–24 h, after which the precipitation of a white powder was observed. The powder was then filtered and characterized with P‐XRD. As shown in Figure 4a, a very good match between the experimental P‐XRD pattern and that simulated from the SC‐XRD data is observed (this is further confirmed by P‐XRD profile fitting, and details are given in Section S5, see Figure S8 and Table S9). TGA analysis on **1‐MeCN^Pwd^** confirms the presence of the solvent, which is released during thermal scanning below 180°C, immediately before the onset of polymer degradation (Figures 4b and S10). The observed mass loss percentage (9.5 ± 0.8%) corresponds to ca. 3 MeCN molecules per formula unit, in reasonable agreement with the results from SC‐XRD measurements. As FT‐IR analysis proved to be unsatisfactory when dealing with the inclusion of MeCN in a previous work [13], MS analysis of the evolved gases was also carried out to unequivocally identify MeCN (according to NIST library) in the sample (see inset in Figure 4b).

**FIGURE 4:**
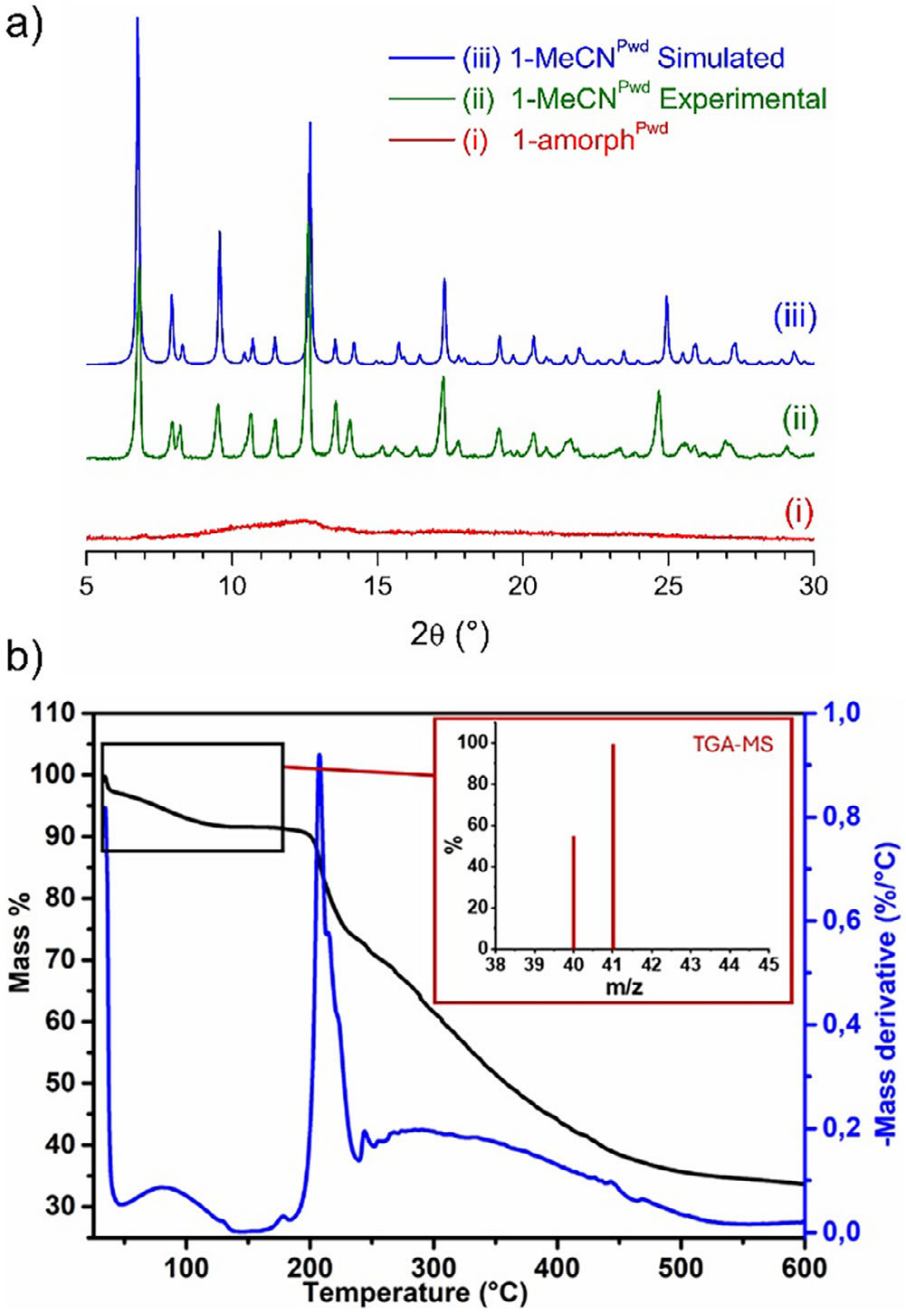
(a) Overlay between P‐XRD patterns of **1‐amorph^Pwd^** obtained upon thermal treatment at 120°C under vacuum (i), the experimental P‐XRD pattern of **1‐MeCN^Pwd^** (ii), and the corresponding simulated from **1‐MeCN^SC^** data (iii). (b) Thermogram of the sample **1‐MeCN^Pwd^**; the inset represents the MS spectrum of the evolved gases. The loss of the MeCN solvent is visible below 180°C, and the degradation of the CP above 180°C.

Vacuum treatment at r.t. for 2 h did not induce significant changes in the P‐XRD pattern of the powder, while more extensive vacuum treatments at 100°C for 5 h led to a loss of crystallinity, which worsens upon temperature increases (up to 120°C), yielding an almost completely amorphous phase (Figure 4a, pattern “i”), which has been named **1‐amorph^Pwd^** thereafter.

### 2.4 Powder Adsorption and Exchange Tests

Exchange experiments with the four VOC solvents previously used with the single‐crystalline phases were also carried out on **1‐MeCN^Pwd^**, and the amorphous phase **1‐amorph^Pwd^** was subsequently tested for adsorption. Exchange experiments carried out over a 4 week time period confirmed that the powders remained crystalline during the whole testing period. As P‐XRD analysis is not very informative due to the similarity of the diffraction patterns of **1‐MeCN^Pwd^** with the final products (Figures S7–S9), TGA‐FT‐IR and TGA‐MS data were also acquired. Figure 5 shows a typical TGA plot (of the sample exchanged with ClBz) recorded in the range 25°C–180°C, with the FTIR and the MS spectra of the gases evolved at around 145°C. The data collected for all the samples are summarized in Table 1, while the thermograms and the spectra of the evolved gases are reported in Figure 5 and in Figures S11–S17. All the samples (except for the ones exposed to Tol vapors) show a small mass loss below 50°C, due to the rapid release of solvent remained on sample surface, and the main solvent loss (with the maximum degradation rate at temperatures at around 175°C for NB, 145°C for ClBz, and 165°C for DCB), preceded by a large shoulder. The thermograms obtained by exposing to NB, ClBz, or DCB vapors **1‐amorph^Pwd^** or **1‐MeCN^Pwd^** are practically equivalent. The samples exposed to Tol vapors, instead, have a different behavior. The one obtained with **1‐amorph^Pwd^** has a single broad mass loss, accounting for a few percentage points (Figure S11); the one obtained with **1‐MeCN^Pwd^** has a profile more similar to the other solvents, with the maximum degradation rate at temperatures around 135°C (Figure S15). Notably, both FT‐IR and MS analyses of the desolvation stream identify in all cases the aromatic solvent (Figures 5 and S11–S17). Mass losses attributable to solvent release, considered in the whole 25°C–180°C range, can be reasonably discriminated from losses due to the degradation of the CP, although in the case of NB, there can be a partial overlap which may result in an underestimation of the solvent contribution. In general, mass losses are found in the 12%–18% range (Table 1), which corresponds to a range of 1.7–2.1 solvent molecules per formula unit (that can be roughly approximated to one solvent molecule per ligand). The only exception is due to the sample obtained by the Tol adsorption, where we observed a significantly lower adsorption compared to other solvents. Indeed, the amount of Tol can be estimated to be ca. 0.9 molecules per formula unit, about half of the value obtained for the other VOCs.

**FIGURE 5:**
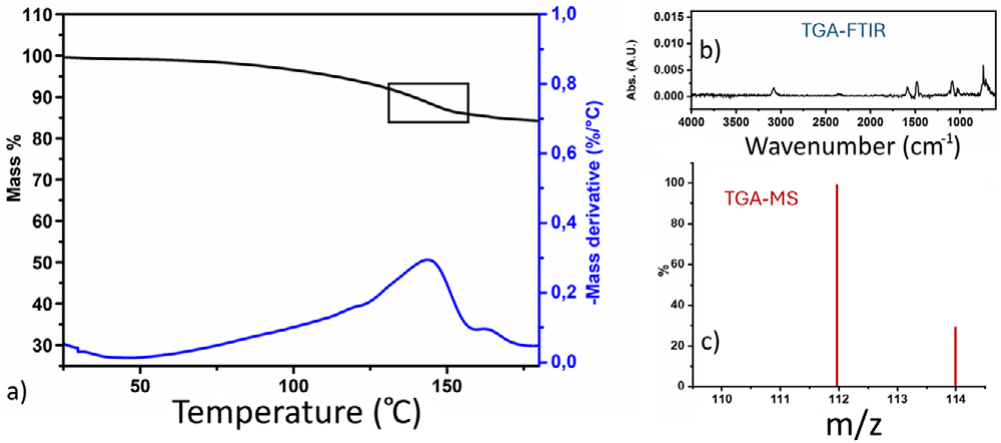
(a) Thermogram of the sample **1‐amorph^Pwd^** after 4 weeks of exposure to ClBz vapors. (b) FT‐IR and (c) MS spectra of the gases evolved at around 145°C. Both spectra are attributed to the solvent ClBz, as confirmed by the NIST library.

**TABLE 1:** Mass loss for each formula unit obtained by TGA measurements and estimated values from SC‐XRD data.

Solvent	Exchange by 1‐MeCN^**Pwd**^		Adsorption by 1‐amorph^**Pwd**^		SC‐XRD data
	Mass loss%	Corresponding solvent molecules per formula unit	Mass loss%	Corresponding solvent molecules per formula unit	Corresponding solvent molecules per formula unit
MeCN	—	—	9.5 ± 0.8	3.0 ± 0.3	4
Tol	12.0 ± 0.4	1.7 ± 0.1	6.6 ± 0.4	0.9 ± 0.1	—
ClBz	16.0 ± 0.2	1.9 ± 0.1	15.7 ± 0.5	1.9 ± 0.1	1.40
DCB	17.4 ± 0.4	1.7 ± 0.1	19.6 ± 0.5	1.9 ± 0.1	0.66
NB	17.8 ± 0.4	2.1 ± 0.1	19.0 ± 0.1	2.2 ± 0.1	2

Formula unit = [Mn(**L1**)_2_Cl_2_]; and solvent amount inferred from s.o.f.

As far as adsorption experiments are concerned, the starting evacuated powder **1‐amorph^Pwd^** was kept within the saturated VOC atmosphere over a period of 8 weeks and characterized over 1 week time intervals for the first 3 weeks and after 2 months. Figure 6 shows a comparison of the P‐XRD data for the starting phase and its evolution toward the final 8 week exposed product phase. Data show that adsorption occurs over different time periods depending on the VOC identity, but in all cases, ultimately, a highly crystalline phase is obtained. Clearly, it can be inferred that adsorption for DCB and Tol is slower, while ClBz is the VOC species that is more quickly adsorbed by **1‐amorph^Pwd^**. Final P‐XRD patterns can be compared with patterns simulated from SC data and with the P‐XRD pattern of the phase obtained by exposure of **1‐MeCN^Pwd^** to the corresponding VOC: the final end products of the two different processes are equivalent in terms of P‐XRD characterization (Figure 6 and S9). Overall, these data show a marked tendency for **1‐amorph^Pwd^** for the adsorption of aromatic VOCs. Data reported in Table 1 can be converted to a more practical mmol/g unit. Thus, exposure of the material to aromatic VOC vapors leads to loadings between ca. 1.6 and 1.9 mmol/g for ClBz, DCB, and NB (corresponding to 1.9–2.2 guest molecules, see Table 1), with a more limited 0.7 mmol/g for Tol. On the other hand, MeCN uptake can be estimated to be ca. 2.5 mmol/g. These values are essentially comparable to those reported for the Cu(II)‐based CPs [13], and they also closely match the loadings observed in earlier bispidine‐based 1D CP adsorbers, for which uptake of small VOCs typically lies in the 1–3 mmol/g interval for near‐saturation vapor exposures [23, 24]. What clearly distinguishes the present Mn(II) system, however, is its adsorption‐induced structural response. Previously reported Mn(II) bispidine CPs feature crystalline architectures built from parallel ribbon‐like arrays, whose relative positions are strongly modulated by the nature and amount of the occluded solvent. As a consequence, changes in the P‐XRD patterns were not only diagnostic of the occurrence of exchange or adsorption, but could also be used as reliable fingerprints of the different solvent‐containing phases. By contrast, in the orthogonally packed Mn(II) CPs described here, regardless of the solvent included, the orientation of the arrays remained unchanged. This aspect can be important for several reasons, as this feature decouples the uptake from large‐scale framework reorganization. If the orthogonal motif is retained, adsorption/exchange likely proceeds via local adjustments rather than macroscopic sliding/reorientation of the ribbons. That represents a different type of adsorption mechanism and structural response than in the parallel ribbon‐like CP analogues. Furthermore, this might also put in question the selectivity of these CP systems, as it could be expected, or proposed, that having the same final CP architecture for the guest‐loaded framework could diminish the host–guest recognition aspects underlying a solvent‐dependent adsorption process. Hence, additional experiments were carried out in order to probe the potential of the **1‐amorph^Pwd^** material for selective adsorption by exposing it for three weeks to two binary vapor mixtures. They were: (i) Tol/NB to test discrimination between two aromatic VOCs differing in substituent electronic and steric features, and (ii) DCB/ClBz to assess whether the number of substituents may influence the uptake. The post‐exposure characterization relied on TGA–MS analysis (Figures S18–S21), which allows for the detection of the molecules released from a sample during the thermal scan [32, 33]. The solvent‐specific single ion current (SIC) signals were considered to deconvolute the contribution of each component. For both mixtures, the thermograms show an initial 1–2 wt% mass loss attributed to superficially adsorbed solvent and containing both mixture components, but the subsequent desorption behavior diverges. To semi‐quantify the internal composition, a calibration experiment was carried out by fully desorbing an equimolar mixture of NB/Tol/ClBz/DCB (0.1 mmol each) adsorbed on alumina under the same thermal program (50°C/min to 180°C, then isothermal to complete evaporation, see Figure S18; relative instrumental response factors are: NB = 1, Tol = 1.8, ClBz = 1.6, and DCB = 1.5). In the Tol/NB case (Figure S19), Tol is released at lower temperatures (viz., earlier times), followed by NB at higher temperatures, indicating stronger retention of NB in the bulk. Data showed that the MS signal associated with NB is ca. 1.1 times that of Tol, thus leading to an estimated ca. 2 NB molecules per 1 Tol molecule in the CP sample based on the response factors obtained from the calibration. It is worth noting that the portion of solvent released at low temperature (i.e., adsorbed on the CP surface) was not included in the integral calculation of the SIC signal. As to the ClBz/DCB mixture (Figure S20), the main release events occur at only slightly different times; data indicate that the DCB signal is ca. 5x the ClBz signal, and considering the similar normalization factors for ClBz and DCB (1.57 vs. 1.5, see Supporting Information), the latter is significantly more abundant (ca. 5x) in the CP. Finally, exposing the material simultaneously to all four solvents (ClBz, DCB, Tol, and NB) for 3 weeks shows desorption steps in which all components are detectable (Figure S21), with DCB released notably more slowly, this being consistent with stronger retention within the CP material. Based on the chromatogram profile (Figure S21) and the response factor calibration (Figure S18), selectivity qualitatively follows this trend: ClBz >> NB > DCB ≈Tol.

**FIGURE 6:**
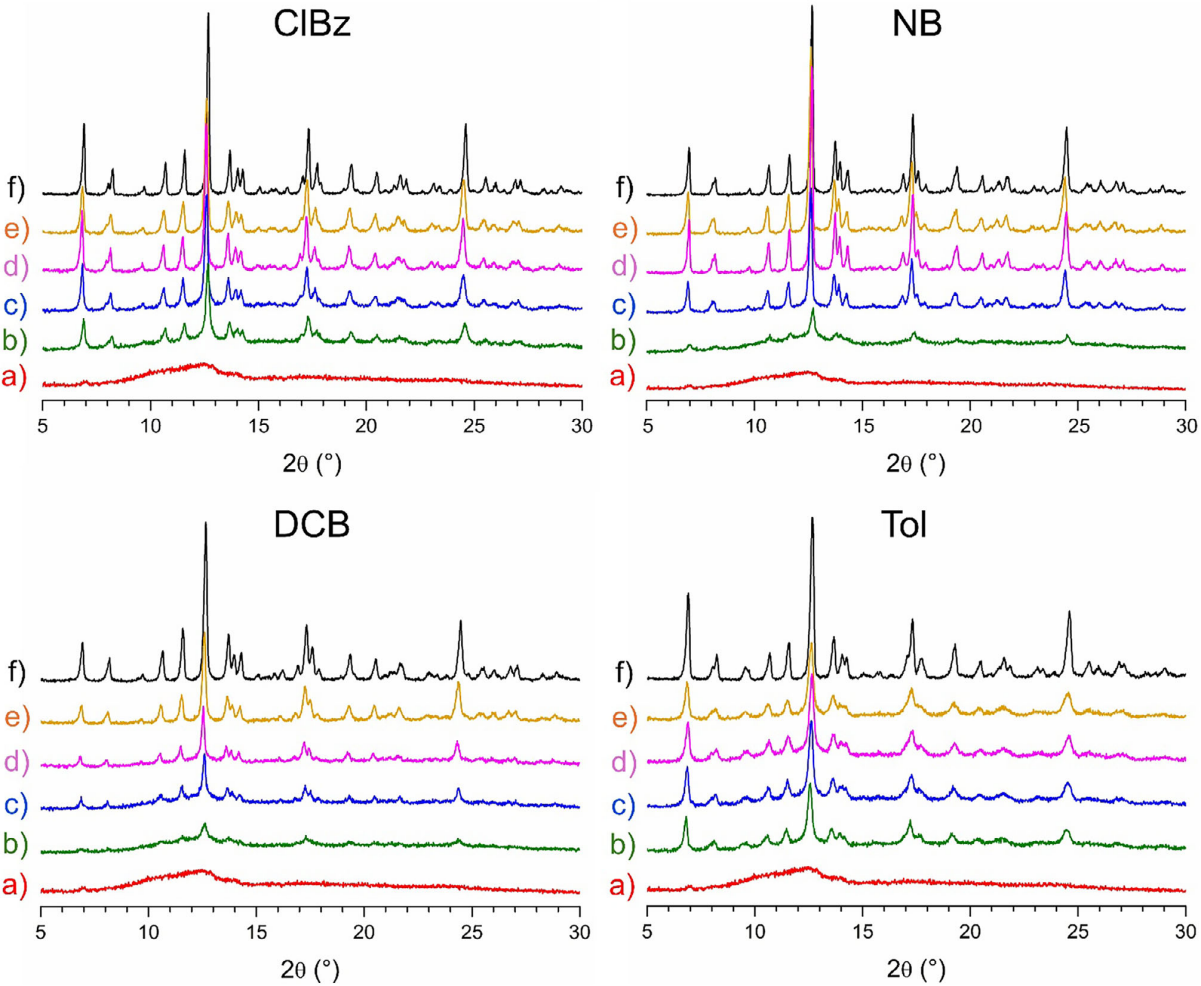
PXRD patterns of 1‐VOC^Pwd^ in four different solvents: VOC = ClBz, NB, DCB, and Tol. In each plot: (a) starting material, **1‐amorph^Pwd^**. (b–e) Patterns recorded after 1, 2, 3, and 8 weeks from the solvent exposure. (f) Reference pattern from the exchange patterns.

## 3 Conclusion

In this study, we report on a new Mn(II)‐bispidine 1D CP, **1‐MeCN**, generated by combining chiral ligand **L1** with MnCl_2_. This CP is characterized by a packing of the ribbon‐like chains in an orthogonal fashion, which is unprecedented within this family of compounds. SC‐XRD analysis of **1‐MeCN^SC^** shows that the polymer crystallizes in the tetragonal space group *P*4_2_2_1_2, with Mn(II) centers in a distorted octahedral {MnN_4_Cl_2_} environment and **L1** acting as a bridging linker to form linear ribbon motifs. Unlike previously reported bispidine‐based Mn(II) 1D CPs, which are typically built from parallel ribbon arrays, this framework assembles into sets of 1D ribbons oriented along orthogonal directions. The resulting packing creates perpendicularly intersecting, wavy 1D channels (ca. 7.5 Å) that account for roughly 30% of the unit‐cell volume upon virtual desolvation. The corresponding powder, **1‐MeCN^Pwd^**, fully characterized by P‐XRD and TGA‐FTIR/MS analysis, is readily accessible by fast precipitation. Solvent exchange experiments demonstrate that this CP framework is functionally capable of SC‐to‐SC transformations, as exposure to saturated vapors of NB, ClBz, and DCB produces new crystalline inclusion phases in which the initial 4 MeCN molecules are replaced by approximately 2.0 NB, 1.4 ClBz, or 0.66 DCB equivalents per formula unit. In contrast, Tol does not undergo an analogous SC‐to‐SC exchange under the same conditions, pointing to a less favorable host–guest match or kinetic constraints. Most importantly, across the successful SC‐to‐SC transformations, the core orthogonal packing motif is retained: the chain orientation and overall framework topology remain essentially the same, indicating that guest exchange proceeds without the substantial ribbon reorganization that characterizes many parallel‐ribbon bispidine CPs. In order to evaluate the CP sorption behavior for potential practical applications, we then tested for adsorption a thermally generated evacuated phase **1‐amorph^Pwd^**, obtained by vacuum treatment at elevated temperature (up to ca. 120°C) of **1‐MeCN^Pwd^**. **1‐amorph^Pwd^** remains indeed an active sorbent and, upon exposure to saturated VOC atmospheres, it evolves back toward highly crystalline phases, as tracked by time‐resolved P‐XRD during multi‐week exposures. Although powder diffraction alone is not diagnostic in this particular CP system due to the strong similarities of the XRD patterns of the various solvent‐containing phases, adsorption assignments are strongly strengthened by coupling P‐XRD with TGA‐FT‐IR and TGA‐MS. Across NB, ClBz, and DCB, thermograms show a small low‐temperature loss (< 50°C) attributable to superficial solvent, followed by the main desorption event (with characteristic maxima around 175°C for NB, 145°C for ClBz, and 165°C for DCB), and the combination of FT‐IR and MS of the evolved gases unambiguously identifies the corresponding analytes. Quantitatively, solvent‐associated mass losses are largely in the 12%–18% range, corresponding to about 1.7–2.1 guest molecules per formula unit (approximately one guest per ligand), whereas toluene is consistently lower (about 0.9 molecules per formula unit) and displays a distinct thermogravimetric profile, consistent with its weaker overall uptake. Converting these loadings to more practical metrics, **1‐amorph^Pwd^** captures roughly 1.6–1.9 mmol/g of NB, ClBz, and DCB under saturated vapor conditions at room temperature (vs. only ca. 0.7 mmol/g for toluene), and shows even higher uptake for MeCN (2.5 mmol/g). These uptakes can be compared to those reported for MOF systems (see Table S10). The most studied VOC is certainly Tol, which is adsorbed in the ca. 1.4 to 6.3 mmol/g range, with the best adsorbents being Ga‐BPT and MIL‐101(Cr) MOFs [34, 35]. ClBz, DCB, and NB are less‐investigated targets, with reported uptakes in the 4–11 mmol/g range. In any case, direct comparisons among these values should be approached with caution due to variations in experimental conditions.

Finally, TGA‐MS selectivity experiments on binary and quaternary vapor mixtures reveal differentiated retention and release behavior. In particular, a stronger retention of NB versus Tol and DCB versus ClBz was observed when binary mixtures were tested. As to quaternary mixtures, the weakest affinity for Tol was confirmed, while a strong preference for ClBz versus both NB and DCB was found. The origin of the mixture‐dependent adsorption profiles, particularly evident for DCB versus ClBz when comparing the binary and quaternary mixture data, remains unclear. This suggests that additional, more quantitative studies are needed to determine the CP's actual selectivity, which nonetheless appears to be present.

In conclusion, this work presents a novel Mn(II) bispidine‐based type of 1D CP constituted by orthogonally packed linear ribbon‐like chains. Although relatively unusual from a structural point of view in comparison with the more classic bispidine‐based CPs, this material represents a functionally relevant modification which reinforces the case for this class of materials as a versatile platform for future development of efficient and selective adsorbers of harmful VOCs operating at r.t. and atmospheric pressure.

## 4 Experimental Section

### 4.1 Materials

The bispidine ligand **L1** (C_30_H_32_N_4_O_5_, 3‐methyl‐7‐(s)‐(−)‐α‐methylbenzyl‐9‐oxo‐2,4‐di(pyridin‐4‐yl)‐3,7‐diazabicyclo[3.3.1]nonane‐1,5‐dicarboxylate) (see Scheme 1) was synthesized following the procedure reported by Sayed and Cametti [36]. Manganese chloride (MnCl_2_, ≥ 99.0%) and all of the solvents: diethyl ether (DEE, ≥ 99.0%), methanol (MeOH, 99.85%), ethanol (EtOH, 99.5%), trichloromethane (TCM, 99.8%), acetonitrile (MeCN, 99.8%), NB (99.0%), CIBz (99.8%), DCB (99.0%), and Tol (≥ 99.5%) were purchased and used as received.

### 4.2 Preparation of **L1** and **1‐MeCN CP**

Single crystals of **L1** (**1^SC^**) were obtained in 24 h by allowing slow diffusion of 1 mL of DEE layered on top of a solution of the ligand (20 mg in 2 mL of EtOH). Single crystals of **1‐MeCN^SC^** were obtained by mixing 7 mL of a MeCN solution of **L1** (0.028 mmol) with 1 mL of a MeOH solution of MnCl_2_ (0.056 mmol). Slow evaporation of the mixture yielded intense yellow prismatic‐pyramidal crystals at the bottom of the vial after a few days.

**1‐MeCN^Pwd^** was prepared using the fast‐crystallization procedure, which involved dissolving 100 mg of **L1** in 3 mL of MeCN. 1 mL of a MeOH solution of MnCl_2_ (2 equivalents) was then carefully added dropwise to the ligand solution while stirring. The resulting mixture was left standing until a pale‐yellow precipitate formed. The precipitate was then collected by filtration and dried (yield, 80%).

### 4.3 Crystallographic Characterization

Single‐crystal x‐ray diffraction (SC‐XRD) studies were performed on ligand **L1** (**1^SC^** in the following) and its Mn‐based CPs (**1‐MeCN^SC^**, **1‐NB^SC^**, **1‐ClBz^SC^**, and **1‐DCB^SC^**), including those as synthesized and post‐adsorption experiments. Data were collected employing a D8 Venture (Bruker AXS) diffractometer operating with an Incoatec microfocus dual source and a Photon III CPAD detector; specifically, Cu *K*_α_ radiation (*λ* = 1.54178 Å) was used for **1^SC^**, while Mo *K*_α_ (*λ* = 0.71073 Å) was used for the Mn‐based CPs. Measurements were conducted at 100(2) K under a continuous nitrogen flow regulated by an Oxford Cryostream 800 Plus cryostat. A combination of *ω*‐ and *ϕ*‐scans with 1.0° frame widths ensured data completeness ≥ 99.5%.

Data integration and multi‐scan absorption correction were carried out using the APEX5 v.2023 suite [37], incorporating SAINT 8.40a and SADABS‐2016/2 tools [37, 38]. Structures were solved via the direct methods with ShelXT v.2018/2 [39], and refined by full‐matrix least‐squares based on *F* ^2^ with ShelXL v.2018/3 [40], implemented in the Olex 2 program [41]. The CPs’ structures exhibit positional disorder, which was carefully modeled (detailed refinements are provided in the Supporting Information). Crystallographic data, along with selected experimental details and refinement parameters, for **1^SC^** are reported in Table S1. For Mn‐CPs, see Table S3. Copies of the crystallographic data can be obtained free of charge on application to the joint Cambridge Crystallographic Data Centre and Fachinformationszentrum Karlsruhe (http://www.ccdc.cam.ac.uk/structures) [42].

All CP structures are described with A‐labeled disorder for the (s)‐(−)‐α‐methylbenzylamino group (s.o.f. = 0.50). In **1‐MeCN^SC^**, the model includes the identified MeCN molecule, while in **1‐ClBz^SC^**, highly occupied solvent sites (1S and 3S with s.o.f. = 0.30) were considered.

P‐XRD data were collected by means of a Bruker AXS D2 PHASER diffractometer equipped with a linear Lynx‐eye position‐sensitive detector (PSD) at aperture 3, anode (Ni‐filtered Cu *K*_α1,2_ radiation). Powder samples were deposited in the hollow of an alumina zero‐background plate, 0.2 mm deep. The acquisition of the diffraction patterns was performed in the 2*θ* range from 4° to 30°, with a sampling interval of 0.02° increments and 0.5 sec/step.

### 4.4 Thermogravimetry (TG) Coupled With FT‐IR or With MS

Thermogravimetry (TG) coupled with FT‐IR analysis of the gases evolved was used to evaluate the type and the amount of VOC desorbed. The instrument used was a Thermobalance Q5000IR (TA Instruments) connected through a transfer line to an FT‐IR Agilent Technologies spectrophotometer (Cary 640). The sample (around 15 mg) was put in a Pt crucible and heated at 10°C/min under nitrogen flow over the temperature range 30°C–600°C. Each sample was analyzed in triplicate. For reasons of time, scanning in replicates was carried out only within the temperature range in which the solvent was desorbed (from 30°C to 180°C). FT‐IR spectra were acquired during the thermal scan. In more detail, a spectrum was recorded every 30 s in the range 400–4000 cm^−1^ with a 4 cm^−1^ width slit. To avoid undesired moisture signals, the optical bench was purged with nitrogen (N_2_), and a background spectrum was recorded before each analysis and automatically subtracted from the recorded spectrum. The identification of the FTIR spectra was performed using the Vapor Phase (Gas) Infrared Spectra of Spectrabase repository (free version) of John Wiley & Sons, Inc. Also, TG coupled with MS spectrometry was used to confirm the identity of VOCs desorbed. The instrument used was a Thermobalance Perkin Elmer TGA8000 connected through a transfer line to a mass spectrometer Perkin Elmer Clarus SQ8T. The sample (around 5 mg) was placed in a Pt crucible and heated at 50°C/min under nitrogen flow in the temperature range 30°C–180°C. Then kept at the final temperature for an additional 5 min. The pyrolysis products were eluted with a helium flow (purity 99.9995%) of 1.0 mL/min. The mass spectrometer operated via electron ionization (EI) at 70 eV and was equipped with a quadrupole operating in positive mode over the mass range of 35–550 *m/z*. The ion source and the quadrupole temperature were set to 250°C. The current profiles were acquired in SIC mode using TurboMass software (version 6.1.2), selecting the two most abundant fragments for each solvent, based on the NIST (National Institute of Standards and Technology, US) spectral libraries. More in detail: NB: *m/z*: 77, 123; Tol: *m/z*: 91, 92; ClBz: *m/z*: 112, 114; DCB: *m/z*: 146, 148.

### 4.5 Solvent‐Exchange Experiments

The solvent‐exchange ability of **1‐MeCN^SC^** with aromatic solvents (NB, ClBz, DCB, and Tol) was investigated using single‐crystal specimens. Prior to the exchange process, the crystals were examined to confirm structural integrity and high crystalline quality. Subsequently, they were placed in a sealed chamber containing a vessel of the respective solvent. After a 2 week exposure period, the crystals were retrieved and characterized by SC‐XRD. Analogous solvent‐exchange experiments were also performed on powder samples, starting from **1‐MeCN^Pwd^**, to evaluate the exchange behavior on a larger scale. The powder samples were analyzed by P‐XRD methods.

### 4.6 Selectivity Experiments

The selectivity of the samples exposed to mixtures of various solvents was investigated using TGA‐MS. First of all, the instrumental response to different ions was registered by recording the signals due to the complete evaporation of a drop of an equimolar mixture of NB/Tol/ClBz/DCB adsorbed on a deactivated alumina powder (inert support). The samples underwent a thermal scan at 50°C/min until 180°C, followed by 15 min of isotherm at 180°C, to enable complete evaporation of the mixture, and the vapors were analyzed by a mass spectrometer operating in the same conditions described above. The SIC signals related to *m/z*: 77, 91, 112, and 146 (Figure S18) were used to obtain the instrumental normalization factors to be used in the selectivity tests. It is worth noting that the signal at 77 was due to fragments belonging to both NB and ClBz, so the latter was subtracted (according to the relative abundance of this peak with respect to the peak at 112), isolating only the NB contribution. In a second moment, the sample **1‐amorph^Pwd^** was exposed for 3 weeks to a mixture of solvent vapors (Mix‐1: Tol/NB; Mix‐2: DCB/ClBz; Mix‐3: Tol/NB/ClBz/DCB). Then, the samples were heated in TGA up to 180°C at 50°C/min and kept at this temperature for a time range between 5 and 15 min. The operating conditions of the MS spectrometer are the same as described in the paragraph above. The peak area, normalized for the factors found as described above, was compared to obtain semiquantitative information on the extent of adsorbed solvents.

## Conflicts of Interest

The author declares no conflicts of interest.

## Supporting information



The authors have cited additional references within the Supporting Information [43, 44, 45, 46, 47, 48, 49, 50, 51]. Supplementary data associated with this work include the crystallographic characterization details and tables for **1^SC^**, and **1‐MeCN^Pwd^**, **1‐NB^Pwd^**, **1‐ClBz^Pwd^** and **1‐DCB^Pwd^** CPs; the structural description of **1^SC^**, additional P‐XRD data for **1‐amorph^Pwd^**, **1‐MeCN^Pwd^**, **1‐NB^Pwd^**, **1‐ClBz^Pwd^** and **1‐DCB^Pwd^** CPs; coupled TGA‐FTIR and TGA‐MS for the adsorption, solvent‐exchange, and selectivity tests. **Supporting File 1**: chem71316‐sup‐0001‐SuppMat.docx

## Data Availability

The data that support the findings of this study are available from the corresponding author upon reasonable request.
