# Supplementary material for: VOCs Adsorption and Exchange Properties in Bispidine‐Based Mn(II) 1D CPs Made of Orthogonally Oriented Linear Chains
Source: Chemistry. 2026 Jul 7;32(39):e71316. doi: 10.1002/chem.71316 (PMC13715454; doi:10.1002/chem.71316)
Supplement: Supplementary file 1 [file CHEM-32-e71316-s001.docx]

**Supporting Information**

VOCs Adsorption and Exchange Properties in Bispidine-Based Mn(II) 1D CPs Made of Orthogonally Oriented Linear Chains

Meriem Goudjil ^[a]^, Martina Lippi *^[a]^, Sara Camorali ^[b]^, Chiara Pelosi ^[c]^, Alessandro Sayed ^[b]^, Luca Bernazzani ^[c][d]^, Patrizia Rossi ^[a]^, Paola Paoli ^[a]^ and Massimo Cametti *^[b]^

[a]	Dr. M. Goudjil, Dr. M. Lippi, Prof. P. Rossi, Prof. P. Paoli

Department of Industrial Engineering

University of Firenze

Via Santa Marta 3, 50139, Firenze, Italy.

E-mail: martina.lippi@unifi.it

[b]	Dr. S. Camorali, Mr. A. Sayed, Prof. M. Cametti

Department of Chemistry, Materials and Chemical Engineering “Giulio Natta”,

Politecnico di Milano

Via Edoardo Bassini 6, 20133, Milano, Italy.

email: [massimo.cametti@polimi.it](mailto:massimo.cametti@polimi.it)

[c]	Dr. C. Pelosi, Prof. L. Bernazzani

Department of Chemistry and Industrial Chemistry

University of Pisa

via G. Moruzzi 13, 56124, Pisa, Italy.

[d]	Prof. L. Bernazzani

Institute for the Chemistry of Organometallic Compounds-ICCOM

Italian National Research Council-CNR

via G. Moruzzi 13, 56124, Pisa, Italy.

**Table of contents**

1. Crystallographic characterization (SC-XRD) ………………………………………………………………. S1

*Refinement details* …………………………………………………………………………………………S1

*Structure solution and refinement parameters for* ***1^SC^*** ………………………………………………...S2

*Structural description of the* ***1^SC^*** …………………………………………………………………………. S2

2. Crystallographic data for the studied Mn(II)-Bispidine CPs ………………………………………………..S6
3. CCDC survey and geometric parameters of Mn(II) Bispidine-based CPs ………………………………..S7
4. Structural projections and intermolecular interactions of Mn(II) CPs ……………………………………. S9
5. P-XRD characterization ……………………………………………………………………………………...S11

*Pattern indexing and Profile fitting* ………………………………………………………………………S11

*Powder adsorption and exchange tests* ………………………………………………………………. S14

6. Thermal analysis of **1-MeCN^Pwd^** ……………………………………………………………………………S15
7. Coupled thermal and spectroscopic analyses …………………………………………………………… S15
8. Literature adsorption data for investigated VOCs…........................................................................... S23

References ……………………………………………………………………………………………………S24

**1. Crystallographic characterization (SC-XRD)**

- *Refinement details*

In the **1^SC^** structure, the asymmetric unit (ASU) comprises one ligand molecule. Non-hydrogen atoms were located in the Fourier difference maps and refined anisotropically. Hydrogen atoms were placed in calculated positions and refined using the riding model. Crystallographic data are given in Table S1.

For the **1-MnCl_2_** CPs, each ASU contains one **L1** molecule and two crystallographically independent Mn atoms occupy special positions: Mn1 at (½,½,½) and Mn2 at (0,0,½), each one with site occupancy factor (s.o.f.) 0.25. Chloride atoms are located at two inequivalent special sites: Cl1 at (−*x+*½,*x+*½,½) and Cl2 at (*x+*½,−*x+*½,½), each one with s.o.f. 0.50. The resulting formula unit is [Mn(**L1**)_2_Cl_2_]_∞_, supplemented by entrapped solvent molecules such as MeCN, NB, ClBz, and DCB (see Table S3). In all CP structures, the (s)-(−)-α-methylbenzylamino group is disordered over two positions (labeled A and B). The structures were described based on A, given its major role within the overall packing. These were refined using PART commands with a fixed s.o.f. = 0.50. Hydrogen atoms were geometrically placed, and their thermal parameters were refined using the riding model.

In **1-MeCN^SC^**, **1-NB^SC^**, and **1-ClBz^SC^** CPs, all non-hydrogen atoms, except those affected by disorder of the (s)-(−)-α-methylbenzylamino group, were refined anisotropically. In **1-MeCN^SC^**, two MeCN molecules are present per ASU: one was located in the Fourier difference maps and refined anisotropically, while the second, disordered, was inferred from the solvent mask. A total of 22 electrons per ASU supports the presence of an additional MeCN molecule. In **1-NB^SC^**, extensive solvent disorder precluded satisfactory modeling; the diffuse electron density was addressed using the solvent mask, revealing the presence of a solvent-accessible void containing 65 e- per ASU, consistent with one NB molecule. A two-component inversion twin was modeled, yielding a refined BASF value of 0.71(4). In the case of **1-ClBz^SC^**, the ClBz solvent exhibits static positional disorder, which was carefully modeled across three positions (labeled as 1S, 2S, and 3S), with s.o.f. set at 0.30, 0.10, and 0.30, respectively. Over the third position, C1SC—Cl3S was fixed to 1.74 Å. Due to the extent of disorder and low occupancy, ClBz atoms were refined isotropically. The structure of **1-ClBz^SC^** is described within solvent molecules with s.o.f. = 0.30. In **1-DCB^SC^**, the presence of DCB was confirmed, with a s.o.f of 0.33 in the ASU. Only the Mn and Cl atoms were refined anisotropically. The final refinement yielded *R*_1_ [*I* ≥ 2*σ* (*I*)] = 0.1263. Minor crystal damage during adsorption likely contributed to reduced data quality and limited the extent of anisotropic refinement.

- *Structure solution and refinement parameters for* ***1^SC^***

**Table S1.** Crystal data and structure refinement parameters for **1^SC^**.

| **Structural parameters** | **1^SC^** |
| --- | --- |
| empirical formula | C_30_H_32_N_4_O_5_ |
| formula weight (g·mol^-1^) | 528.59 |
| temperature (K) | 100(2) |
| radiation (wavelength, Å) | Cu *K*_α_ (λ = 1.54178) |
| crystal system | orthorhombic |
| space group | *P*2_1_2_1_2_1_ |
| *a* (Å) | 9.6022(4) |
| *b* (Å) | 14.1738(7) |
| *c* (Å) | 19.4330(9) |
| volume (Å^3^) | 2644.8(2) |
| *Z* | 4 |
| ρ_calc_ (mg·m^-3^) | 1.328 |
| crystal size (mm) | 0.36 × 0.25 × 0.04 |
| crystal color/shape | yellowish/block |
| μ (mm^-1^) | 0.746 |
| 2θ range (°) | 7.72-136.75 |
| *F*(000) | 1120 |
| *hkl* range | -11 ≤ *h* ≤ 11 |
|  | -16 ≤ *k* ≤ 17 |
|  | -23 ≤ *l* ≤ 23 |
| no. of reflections collected | 23424 |
| no. of independent reflections | 4834 |
| data/restraints/parameters | 4834/0/356 |
| *R*_1_ [*I* ≥ 2*σ* (*I*)] | 0.0362 |
| *wR*_2_ [*I* ≥ 2*σ* (*I*)] | 0.0920 |
| GooF on *F*^2^ | 1.052 |
| largest difference map hole | Δρ_min_ = −0.25 e·Å^–3^; Δρ_max_ = 0.24 e·Å^–3^ |

- *Structural description of the* ***1^SC^***

The **L1** ligand crystallizes in the orthorhombic space group *P*2_1_2_1_2_1_. In bispidine ligands, ester groups can exhibit distinct orientation with respect to each other; in **1^SC^** they are *syn*-oriented (Fig.S1.a). Within the crystal lattice, each molecule interacts with eight adjacent molecules, all of which are symmetry-related through operations involving the 2_1_ screw axes, see Fig.S1.b.

Along the *a*-axis direction, the **L1** molecule forms four moderate hydrogen bonds with two neighboring molecules (see Table S2): it participates in two identical O···H interactions~~,~~ wherein the aromatic pyridine ring (C9C10C11C12C13N3) acts as a donor to the keto oxygen atom O3 (C12—H12···O3: 2.433(2) Å, 158(1)°; see Fig.S2.a, Fig.S3.b), in addition to two N···H hydrogen bonds are observed, involving the methyl (C21H_3_) group donating to the pyridine nitrogen atom N3 (C21—H21A···N3: 2.447(2) Å, 159(2)°; see Fig.S2.a, Fig. S3.b). This interaction-pattern yields a $R_{2}^{2}(10)$ ring motif.

**Figure S1.** a) Asymmetric unit of **1^SC^** with ellipsoids drawn at 50% probability, ester groups are *syn*-oriented (hydrogen atoms are omitted for clarity); b) Intermolecular contacts between the motif of ligand **L1**, and neighboring molecules related via the 2_1_ screw axes.

Along the *b*-axis direction, **L1** engages in weaker O···H contacts with two neighboring molecules. These contacts occur between the methyl hydrogen H7C and the ester oxygen O4 (C7—H7C···O4: 2.680(2) Å, 135(1)°; Fig.S2.b and Fig.S3.a). One interaction involves **L1** acting as a hydrogen donor, while the other involves it serving as a hydrogen acceptor.

In the *c*-axis direction, ligand **L1** establishes further intermolecular contacts with four molecules. On one side, a bifurcated interaction is observed, where the ester oxygen atom O5 acts as an acceptor for two hydrogen atoms from distinct adjacent molecules: the pyridine hydrogen H1 from one molecule (C1—H1···O5: 2.665(2) Å, 142(1)°; Fig.S2.c and Fig.S3.a) and the methyl hydrogen H23A from another (C23—H23A···O5: 2.577(2) Å, 151(2)°; Fig.S3.b). On the opposite side, hydrogen atoms H1 and H23A interact with two additional neighboring molecules, thereby completing the hydrogen-bonding pattern along the *c*-axis. Furthermore, N···H interactions are present, involving nitrogen N1 and two hydrogen atoms from the same neighboring molecule: one robust from the bispidine core (C17—H17B···N1: 2.710(2) Å, 162(1)°; see Fig.S2.c and Fig.S3.a) and one weak from the methyl (C24—H24A···N1: 2.710(2) Å, 128(1)°; Fig.S2.c) forming an $R_{2}^{2}(8)$ ring motif.

**Figure S2.** Hydrogen bonding interactions in **1^SC^** between **L1** molecules related via the screw axes 2_1_: a) C12—H12···O3 and C21—H21A···N3 along the *a*-axis direction; b) C7—H7C···O4 long the *b*-axis direction; c) C1—H1···O5, C23—H23A···O5, C17—H17B···N1 and C24—H24A···N1 along the *c*-axis direction.

The stacking of the **L1** molecules gives rise to a columnar arrangement along the [100] direction, characterized by layers that slightly interdigitate along [001] (Fig.S3.a).

**Figure S3.** Crystal packing views of **1^SC^** showing the H-bonding interactions: a) C1—H1∙∙∙O5, C7—H7C∙∙∙O4 and C17—H17B∙∙∙N1 along [100]; b) C12—H12∙∙∙O3, C23—H23A∙∙∙O5 and C21—H21A∙∙∙N3 along [010].

**Table S2.** Hydrogen bond geometry (Å,°) in **1^SC^**. Intermolecular distances were evaluated based on the vdW radii sums (H···O ≤ 2.72 Å, H···N ≤ 2.75 Å),^43^ and angle > 120°.

| **D—H···A** | **D—A (Å)** | **H···A (Å)** | **D—H···A (°)** | **Symmetry operation** |
| --- | --- | --- | --- | --- |
| C1—H1···O5 | 3.464(3) | 2.665(2) | 142(2) | -x+3/2,-y+1,+z-1/2 |
| C7—H7C···O4 | 3.448(3) | 2.680(2) | 135(1) | x+1/2,-y+1/2,-z+1 |
| C12—H12···O3 | 3.336(3) | 2.433(2) | 158(2) | x+1,+y,+z |
| C17—H17B···N1 | 3.664(3) | 2.710(2) | 162(1) | -x+3/2,-y+1,+z+1/2 |
| C21—H21A···N3 | 3.383(3) | 2.447(2) | 159(2) | x-1,+y,+z |
| C23—H23A···O5 | 3.444(3) | 2.557(2) | 151(2) | -x+1/2,-y+1,+z-1/2 |
| C24—H24A···N1 | 3.405(3) | 2.710(2) | 128(1) | -x+3/2,-y+1,+z+1/2 |

**2. Crystallographic data of the studied Mn(II)-Bispidine CPs**

**Table S3.** Crystal data, experimental details, and structure refinement parameters for Mn-Bispidine CPs.

| **Structural parameters** | **1-MeCN^SC^** | **1-NB^SC^** | **1-ClBz^SC^** | **1-DCB^SC^** |
| --- | --- | --- | --- | --- |
| empirical formula | Mn(C_30_H_32_N_4_O_5_)_2_Cl_2_·4(C_2_H_3_N) | Mn(C_30_H_32_N_4_O_5_)_2_Cl_2_·2(C_6_H_5_NO_2_) | Mn(C_30_H_32_N_4_O_5_)_2_Cl_2_·1.4(C_6_H_5_Cl) | Mn(C_30_H_32_N_4_O_5_)_2_Cl_2_·0.66(C_6_H_4_Cl_2_) |
| formula weight (g·mol^-1^) | 1347.24 | 1429.25 | 1340.60 | 1280.04 |
| temperature (K) | 100(2) | 100(2) | 100(2) | 100(2) |
| radiation (wavelength, Å) | Mo *K*_α_ (λ = 0.71073) | Mo *K*_α_ (λ = 0.71073) | Mo *K*_α_ (λ = 0.71073) | Mo *K*_α_ (λ = 0.71073) |
| crystal system | tetragonal | tetragonal | tetragonal | tetragonal |
| space group | *P*4_2_2_1_2 | *P*4_2_2_1_2 | *P*4_2_2_1_2 | *P*4_2_2_1_2 |
| *a* (Å) | 18.4785(3) | 18.4163(8) | 18.4424(5) | 18.438(7) |
| *b* (Å) | 18.4785(3) | 18.4163(8) | 18.4424(5) | 18.438(7) |
| *c* (Å) | 21.2867(5) | 21.6869(15) | 21.5195(5) | 21.525(9) |
| volume (Å^3^) | 7268.4(3) | 7355.3(8) | 7319.3(4) | 7318(6) |
| *Z* | 4 | 4 | 4 | 4 |
| ρ_calc_ (mg·m^-3^) | 1.231 | 1.290 | 1.217 | 1.163 |
| crystal size (mm) | 0.12 × 0.12 × 0.06 | 0.28 × 0.22 × 0.12 | 0.38 × 0.14 × 0.09 | 0.26 × 0.22 × 0.12 |
| crystal color/shape | yellow/block | yellow/block | yellow/block | yellow/block |
| μ (mm^-1^) | 0.317 | 0.321 | 0.363 | 0.357 |
| 2θ range (°) | 4.40-56.68 | 4.42-61.18 | 4.41-61.09 | 3.65-61.57 |
| *F*(000) | 2828 | 2988 | 2801 | 2673 |
| *hkl* range | -24 ≤ *h* ≤ 24 | -25 ≤ *h* ≤ 26 | -26 ≤ *h* ≤ 23 | -26 ≤ *h* ≤ 26 |
|  | -23 ≤ *k* ≤ 24 | -26 ≤ *k* ≤ 26 | -26 ≤ *k* ≤ 26 | -26 ≤ *k* ≤ 26 |
|  | -28 ≤ *l* ≤ 28 | -31 ≤ *l* ≤ 30 | -30 ≤ *l* ≤ 30 | -30 ≤ *l* ≤ 30 |
| no. of reflections collected | 191009 | 290942 | 184658 | 114001 |
| no. of independent reflections | 9063 | 11293 | 11220 | 11326 |
| data/restraints/parameters | 9063/0/368 | 11293/0/329 | 11220/1/388 | 11326/0/210 |
| *R*_1_ [*I* ≥ 2*σ* (*I*)] | 0.0418 | 0.0549 | 0.0564 | 0.1263 |
| *wR*_2_ [*I* ≥ 2*σ* (*I*)] | 0.1037 | 0.1524 | 0.1560 | 0.2876 |
| GooF on *F*^2^ | 1.031 | 1.041 | 1.034 | 1.042 |
| largest difference map hole | Δρ_min_ = −0.33 e·Å^–3^;  Δρ_max_ = 0.32 e·Å^–3^ | Δρ_min_ = −0.60 e·Å^–3^;  Δρ_max_ = 0.74 e·Å^–3^ | Δρ_min_ = −0.48 e·Å^–3^;  Δρ_max_ = 0.98 e·Å^–3^ | Δρ_min_ = −0.80 e·Å^–3^;  Δρ_max_ = 0.91 e·Å^–3^ |

**3. CCDC survey and geometric parameters of Mn(II) Bispidine-based CPs**

**Table S4.** Selected details and geometrical parameters (Å) for reported bispidine-based CPs with MnCl_2_ compared to the present study.

| CP | Solvent | SG | Mn—N (Å) | Mn—Cl (Å) | Mn···Mn (Å) | %*V*_voids_ | Reference |
| --- | --- | --- | --- | --- | --- | --- | --- |
| RIMXUJ | NB | *P*-1 | 2.313(2)-2.336(2) | 2.527(1) | 11.83 | 36.0 | ^9^ |
| RIMYAQ | TCM | *P*-1 | 2.330(5)-2.353(6) | 2.460(2)-2.484(2) | 12.84 | 21.3 |  |
| RIMZOF | NB | *P*2_1_/c | 2.354(7) | 2.462(2) | 13.04 | 19.4 |  |
| RIMZUL | TCM | *P*-1 | 2.324(11) | 2.482(5) | 13.05 | 33.0 |  |
| LORKIP | -- | *P*-1 | 2.353(1) | 2.427(1) | 12.99 | 8.8 | ^10^ |
| LORKOV | TCM-p-ClTol | *Pnma* | 2.333(5)-2.353(5) | 2.473(3)-2.477(3) | 13.12 | 17.2 |  |
| LORKUB | TCM-NB | *Pnma* | 2.346(3)-2.378(3) | 2.433(3)-2.475(2) | 13.16 | 19.2 |  |
| LABQEO* | TCM | *P*-1 | 2.346(6) | 2.498(2) | 13.00 | 30.3 | ^11^ |
| LABQIS* | TCM (2 pfu) | *P*-1 | 2.305(4)-2.320(4) | 2.468(2)-2.483(2) | 12.84 | 19.2 |  |
| LABQIS01 | TCM (0.7 pfu) | *P*-1 | 2.326(2)-2.330(2) | 2.473(1) | 12.84 | 21.7 |  |
| LABQUE** | TCM-NB | *P*-1 | 2.310(3) | 2.465(1) | 13.03 | 29.9 |  |
| LABRAL | Me-NB | *P*2_1_/c | 2.137(6) | 2.380(6) | 12.34 | 21.7 |  |
| **1-MeCN^SC^** | MeCN | *P*4_2_2_1_2 | 2.281(3)-2.295(3) | 2.502(3)-2.505(3) | 13.06(1) | 28.7 | This work |
| **1-NB^SC^** | NB |  | 2.291(3)-2.309(3) | 2.483(1)-2.507(1) | 13.02(1) | 29.9 |  |
| **1-ClBz^SC^** | ClBz |  | 2.280(3)-2.312(3) | 2.482(1)-2.489(2) | 13.04(1) | 29.1 |  |
| **1-DCB^SC^** | 1,2-DCB |  | 2.284(12)-2.306(9) | 2.490(2)-2.495(4) | 13.03(2) | 29.8 |  |

*, **: Low temperature (*: *T* = 150 K and **: *T* = 100 K).

**Figure S4.** Comparison of the ribbon chains orientation in **1-MeCN^SC^** (center) and all other Mn(II) Bispidine-based CPs reported so far. In **1-MeCN^SC^**, the chains are orthogonal to each other.

**4. Structural projections and intermolecular interactions of Mn(II) CPs**

**Figure S5.** Packing diagram of **1-MeCN^SC^** viewed along [001] direction, illustrating the arrangement of ordered acetonitrile solvent molecules and the 4_2_-screw axis (blue squares) symmetry relating the 1D chains.

**Table S5.** Hydrogen bond geometry (Å,°) in **1-MeCN^SC^**. Intermolecular distances were evaluated based on the vdW radii sums (H···O ≤ 2.72 Å, H···N ≤ 2.75 Å, H···Cl ≤ 2.95 Å),^43^ and angle > 120°.

| **D—H···A** | **D—A (Å)** | **H···A (Å)** | **D—H···A (°)** | **Symmetry operation** |
| --- | --- | --- | --- | --- |
| C4—H4···O5 | 3.113(4) | 2.477(3) | 124(1) | -x+1/2,+y+1/2,-z+3/2 |
| C5—H5···O5 | 3.169(4) | 2.576(3) | 120(1) | -x+1/2,+y+1/2,-z+3/2 |
| C10—H10···O2 | 3.143(4) | 2.520(2) | 123(1) | x-1/2,-y+1/2,-z+3/2 |
| C11—H11···O2 | 3.178(4) | 2.559(3) | 122(1) | x-1/2,-y+1/2,-z+3/2 |
| C21—H21C···Cl1 | 3.902(4) | 2.942(0) | 166(1) | x-1/2,-y+1/2,-z+3/2 |
| C23—H23B···Cl2 | 3.827(4) | 2.896(0) | 159(1) | y+1/2,-y+1/2,+z+1/2 |
| C2A—H2AB···Cl2 | 3.590(6) | 2.896(1) | 128(1) | y+1,+x,-z+1 |
| C24A—H24C···N1A | 3.551(10) | 2.630(4) | 156(1) | -x+1,-y,+z |

**Table S6.** Hydrogen bond geometry (Å,°) in **1-NB^SC^**. Intermolecular distances were evaluated based on the vdW radii sums (H···O ≤ 2.72 Å, H···N ≤ 2.75 Å, H···Cl ≤ 2.95 Å),^43^ and angle > 120°. Longer H···Cl contacts are included for comparison with other CP structures.

| **D—H···A** | **D—A (Å)** | **H···A (Å)** | **D—H···A (°)** | **Symmetry operation** |
| --- | --- | --- | --- | --- |
| C4—H4···O5 | 3.112(5) | 2.474(3) | 124(1) | -x+3/2,+y+1/2,-z+1/2 |
| C5—H5···O5 | 3.217(5) | 2.628(3) | 120(1) | -x+3/2,+y+1/2,-z+1/2 |
| C10—H10···O2 | 3.162(4) | 2.508(2) | 126(1) | x+1/2,-y+1/2,-z+1/2 |
| C11—H11···O2 | 3.216(4) | 2.625(3) | 120(1) | x+1/2,-y+1/2,-z+1/2 |
| C21—H21C···Cl1 | 3.990(4) | 3.039(1) | 163(1) | -1/2+x,1/2-y,1/2-z |
| C23—H23B···Cl2 | 3.954(3) | 3.027(2) | 158(1) | 3/2-x,-1/2+y,1/2-z |

**Figure S6.** Crystal packing views of a) **1-ClBz^SC^** with ClBz displayed at site 1S, s.o.f. = 0.30 and b) **1-DCB^SC^**, s.o.f. = 0.33, CPs along the [-110]. In both CPs, the entrapped VOCs occupy almost the same space in the polymeric framework as for **1-MeCN^SC^** (see Fig.1b).

**Table S7.** Hydrogen bond geometry (Å,°) in **1-ClBz^SC^**. Intermolecular distances were evaluated based on the vdW radii sums (H···O ≤ 2.72 Å, H···N ≤ 2.75 Å, H···Cl ≤ 2.95 Å),^43^ and angle > 120°. Longer H···Cl contacts are included for comparison with other CP structures.

| **D—H···A** | **D—A (Å)** | | **H···A (Å)** | | **D—H···A (°)** | | **Symmetry operation** | |  |
| --- | --- | --- | --- | --- | --- | --- | --- | --- | --- |
| C4—H4···O5 | | 3.117(5) | | 2.485(3) | | 123(1) | | x-1/2,-y+3/2,-z+1/2 | |
| C5—H5···O5 | | 3.181(5) | | 2.585(3) | | 121(0) | | x-1/2,-y+3/2,-z+1/2 | |
| C10—H10···O2 | | 3.156(5) | | 2.509(3) | | 125(1) | | -x+3/2,+y+1/2,-z+1/2 | |
| C11—H11···O2 | | 3.214(5) | | 2.613(3) | | 121(1) | | -x+3/2,+y+1/2,-z+1/2 | |
| C21—H21C···Cl1 | | 3.960(4) | | 3.009(4) | | 164(1) | | 3/2-x,-1/2+y,1/2-z | |
| C23—H23B···Cl2 | | 3.895(5) | | 2.964(0) | | 158(1) | | x-1/2,-y+3/2,-z+1/2 | |
| C4SA—H4SA···Cl1 | | 3.612(8) | | 2.862(1) | | 136(1) | | x,y,z | |
| C2SA—H2SA···Cl2 | | 3.594(8) | | 2.918(1) | | 129(1) | | -x+2,-y+1,+z | |

**Table S8.** Hydrogen bond geometry (Å,°) in **1-DCB^SC^**. Intermolecular distances were evaluated based on the vdW radii sums (H···O ≤ 2.72 Å, H···N ≤ 2.75 Å, H···Cl ≤ 2.95 Å),^43^ and angle > 120°. Longer H···Cl contacts are included for comparison with other CP structures.

| **D—H···A** | **D—A (Å)** | **H···A (Å)** | **D—H···A (°)** | **Symmetry operation** |
| --- | --- | --- | --- | --- |
| C4—H4···O5 | 3.163(15) | 2.547(9) | 122(1) | x+1/2,-y+3/2,-z+1/2 |
| C5—H5···O5 | 3.210(14) | 2.606(9) | 121(1) | x+1/2,-y+3/2,-z+1/2 |
| C10—H10···O2 | 3.140(17) | 2.498(10) | 125(1) | -x+3/2,+y-1/2,-z+1/2 |
| C11—H11···O2 | 3.185(18) | 2.576(10) | 122(1) | -x+3/2,+y-1/2,-z+1/2 |
| C21—H21C···Cl1 | 3.986(15) | 3.037(2) | 163(1) | 3/2-x,1/2+y,1/2-z |
| C23—H23B···Cl2 | 3.883(15) | 2.948(2) | 159(2) | x+1/2,-y+3/2,-z+1/2 |
| C29A—H29A···Cl2S | 3.605(25) | 2.839(16) | 138(2) | -y+3/2,+x+1/2,+z+1/2 |
| C5S—H5S···Cl2 | 3.672(29) | 2.909(4) | 138(2) | x+1/2,-y+3/2,-z+1/2 |

**5. P-XRD characterization**

- *Pattern indexing and Profile fitting*

P-XRD data were refined with the Pawley method using the TOPAS program.^44^ The background was modeled with a fifth-order Chebyshev polynomial, while peak profiles were described using the pseudo-Voigt Thompson–Cox–Hastings (TC-PVHZ) function. The refinement included lattice parameters, scale factors, peak-shape parameters, and zero-point correction.

The Pawley refinement demonstrates good agreement between the experimental diffraction pattern of the synthesized **1-MeCN^Pwd^** sample and the calculated pattern based on the **1-MeCN^SC^** structural model, see Tab.S9 and Fig.S7. While the patterns are consistent with the tetragonal phase, all P-XRD profiles of the CPs containing aromatic solvents, obtained from adsorption and solvent-exchange experiments, exhibit similar diffraction features. This similarity indicates that the CP framework remains largely intact, with only minor structural rearrangements, as confirmed by SC-XRD; however, minor variations are present. Since the diffraction patterns of all aromatic-solvent CPs are comparable, the **1-ClBz^Pwd^** sample (after 8 weeks of adsorption) is presented here as a representative example. A brief comparison between the P-XRD patterns of **1-MeCN^Pwd^** and **1-ClBz^Pwd^** is provided below.

Compared with **1-MeCN^Pwd^**, the experimental diffraction peaks of the **1-ClBz^Pwd^** at 2*θ* of 6.77° and 9.35°, corresponding respectively to the planes (110) and (102) along which solvent molecules are segregated, exhibit markedly lower intensities (in accordance with the calculated), see Fig.S8. This may arise from subtle preferred-orientation effects introduced by the ClBz substituent solvent. In addition, the peaks at 13.76° and 24.42°, associated with the (113) and (225) planes, respectively, are shifted toward lower angles relative to the calculated pattern, which is explained by the expansion primarily along the *c*-axis (Tab.S9).

**Table S9.** Refined unit-cell parameters and P-XRD refinement fitting metrics

for **1-MeCN^Pwd^** and **1-ClBz^Pwd^**.

| CP Phase | **1-MeCN^Pwd^** | **1-ClBz^Pwd^** (week 8) |
| --- | --- | --- |
| Crystal system | Tetragonal | |
| Space group | *P*4_2_2_1_2 | |
| Lattice parameters (Å) |  |  |
| *a* = *b* | 18.556(3) | 18.546(4) |
| *c* | 21.713(4) | 21.954(5) |
| *Rexp* | 3.98 | 4.98 |
| *Rwp* | 9.66 | 7.66 |
| *Rp* | 7.10 | 5.96 |
| *GOF* | 2.43 | 1.54 |
| *R_Bragg_* | 1.190 | 0.548 |
| *DW* | 0.55 | 1.13 |

*DW: weighted Durbin-Watson parameter.*

**Figure S7.** Refined P-XRD profile for **1-MeCN^Pwd^**.

**Figure S8.** Refined P-XRD profile for **1-ClBz^Pwd^** obtained from adsorption after 8 weeks.

- *Powder adsorption and exchange tests*

**Figure S9**. Comparison between experimental P-XRD pattern of **1-MeCN^Pwd^** (black) with P-XRD patterns of **1-ClBz^Pwd^**, **1-DCB^Pwd^**, **1-NB^Pwd^**, and **1-Tol^Pwd^** obtained from adsorption of VOCs on **1-amorph^Pwd^** (red) and from solvent exchange on **1-MeCN^Pwd^** (blue) after 8 weeks of exposure.

**6. Thermal analysis of 1-MeCN^Pwd^**

**Figure S10.** Additional thermogram of the sample **1-MeCN^Pwd^**. The observed weight loss of ca. 9.48% is consistent with the presence of 4 co-crystallized MeCN molecules per formula unit of the Mn(II)-CP.

**7. Coupled thermal and spectroscopic analyses**

**Figure S11.** Thermogram of the sample **1-amorph^Pwd^** after 4 weeks of exposure to Tol vapors. The inserts are the FTIR (on the top) and MS (on the bottom) spectra of the gases evolved at 100-150°C. Both spectra are attributed to the solvent Tol, as confirmed by the NIST library.

**Figure S12.** Thermogram of the sample **1-amorph^Pwd^** after 4 weeks of exposure to DCB vapors. The inserts are the FTIR (on the top) and MS (on the bottom) spectra of the gases evolved at around 160°C. Both spectra are attributed to the solvent DCB, as confirmed by the NIST library.

**Figure S13.** Thermogram of the sample **1-amorph^Pwd^** after 4 weeks exposure to NB vapors. The inserts are the FTIR (on the top) and MS (on the bottom) spectra of the gases evolved at around 160°C. Both spectra are attributed to the solvent NB, as confirmed by the NIST library.

**Figure S14.** Thermogram of the sample **1-MeCN^Pwd^** after 4 weeks exposure to ClBz vapors. The inserts are the FTIR (on the top) and MS (on the bottom) spectra of the gases evolved at around 150°C. Both spectra are attributed to the solvent ClBz as confirmed by the NIST library.

**Figure S15.** Thermogram of the sample **1-MeCN^Pwd^** after 4 weeks exposure to Tol vapors. The inserts are the FTIR (on the top) and MS (on the bottom) spectra of the gases evolved at 100-150°C. Both spectra are attributed to the solvent Tol, as confirmed by the NIST library.

**Figure S16.** Thermogram of the sample **1-MeCN^Pwd^** after 4 weeks exposure to DCB vapors. The inserts are the FTIR (on the top) and MS (on the bottom) spectra of the gases evolved around 160°C. Both spectra are attributed to the solvent DCB, as confirmed by the NIST library.

**Figure S17.** Thermogram of the sample **1-MeCN^Pwd^** after 4 weeks of exposure to NB vapors. The inserts are the FTIR (on the top) and MS (on the bottom) spectra of the gases evolved at around 160°C. Both spectra are attributed to the solvent NB, as confirmed by the NIST library.

| **Solvent** | **Peak area** | **Corrected peak area** | **ratio** |
| --- | --- | --- | --- |
| NB (m/z = 77) | 4.02E+07 | 2.29E+07 | 1.00 |
| Tol | 4.17E+07 | 4.17E+07 | 1.82 |
| ClBz | 3.59E+07 | 3.59E+07 | 1.57 |
| DCB | 3.44E+07 | 3.44E+07 | 1.50 |

**Figure S18.** Evolution profile of the fragments with m/z: 77, 91, 112, 146 evolved during the evaporation of a drop of an equimolar mixture of NB/Tol/ClBz/DCB adsorbed on an alumina powder. The fragments are related to the following solvents: m/z:77 (red) NB (after subtraction of ClBz contribution); m/z: 91 (blue) Tol; m/z: 112 (purple): ClBz; m/z: 146 (green) DCB).

| **Solvent** | **Peak area** | **Normalized peak area** | **ratio** |
| --- | --- | --- | --- |
| NB (m/z = 77) | 672161.01 | 672161.0113 | 2.05 |
| Tol | 595354.50 | 327456.0412 | 1.00 |

**Figure S19**. Evolution profile of the fragments with m/z: 77, 91 evolved during the thermal scan of **1-amorph^Pwd^** after 3-weeks exposure to vapors of NB/Tol. The fragments are related to the following solvents: m/z:77 (red) NB; m/z: 91 (blue) Tol. The area was normalized for the normalizing factor found as described in the experimental section. The final piece of NB area was reconstructed through a fitting procedure using a gaussian Mod. Function.

| **Solvent** | **Peak area** | **Normalized peak area** | **Ratio** |
| --- | --- | --- | --- |
| ClBz | 3.67E+06 | 2.34E+06 | 1.00 |
| DCB | 1.83E+07 | 8.40E+06 | 3.59 |

**Figure S20.** Evolution profile of the fragments with m/z: 112, 146 evolved during the thermal scan of **1-amorph^Pwd^** after 3-weeks exposure to vapors of ClBz/DCB. The fragments are related to the following solvents: m/z: 112 (purple): ClBz; m/z: 146 (green) DCB. The area was normalized for the normalizing factor found as described in the experimental section. The peaks were reconstructed through a fitting procedure using a gaussian Mod. Function.

| **Solvent** | **Peak area** | **Normalized peak area** | **ratio** | **ratio** |
| --- | --- | --- | --- | --- |
| NB (m/z = 77) | 3.94E+06 | 9.02E+05 | 1.68 | 1.42 |
| Tol | 975656.586 | 5.37E+05 | 1.00 | 0.85 |
| ClBz | 6.33E+06 | 4.04E+06 | 7.53 | 6.38 |
| DCB | 950525.216 | 6.34E+05 | 1.18 | 1.00 |

**Figure S21.** Evolution profile of the fragments with m/z: 77, 91, 112, 146 evolved during the thermal scan of **1-amorph^Pwd^** after 3-weeks exposure to vapors of NB/Tol/ClBz/DCB. The fragments are related to the following solvents: m/z:77 (red) NB (after subtraction of ClBz contribution); m/z: 91 (blue) Tol; m/z: 112 (purple): ClBz; m/z: 146 (green) DCB. The area was normalized for the normalizing factor found as described in the experimental section.

8. **Literature adsorption data for investigated VOCs**

**Table S10.** Comparison of solvent uptake capacities (mmol/g) for representative porous CP materials from the literature. Abbreviations: MeCN = acetonitrile, Tol = toluene, ClBz = chlorobenzene, DCB = dichlorobenzene, and NB = nitrobenzene.

| **Solvent** | **CP / MOF species** | **Uptake mmol/g** | **Ref** |
| --- | --- | --- | --- |
| **MeCN** | Co-MOF-184 | 24.3 | 45 |
|  | SU-102-Mg | 8.4 | 46 |
|  | X-pcu-11-Zn | 8.66 | 47 |
| **Tol** | Ga-BPT MOF | 4.98-6.29 | 48  and references therein |
|  | UiO-66 | 3.22 |  |
|  | HKUST-1 | 4.36 |  |
|  | BUT-66 | 1.36 |  |
|  | ZJU-620 | 2.0 |  |
| **ClBz** | modified UiO-66 | 4.94 | 49 |
| **DCB** | MIL-101(Cr) | 11.04 | 50 |
| **NB** | (sql-1-Co-NCS·2NB) | 4.10 | 51 |

**References**

[9]	Rossetti, A.; Lippi, M.; Martí‐Rujas, J.; Sacchetti, A.; Cametti, M, “Highly Dynamic and Tunable Behavior of 1D Coordination Polymers Based on the Bispidine Ligand” *Chemistry–A European Journal* **24** (72), (2018): 19368–19372.

[10]	Lippi, M.; Cametti, M.; Martí-Rujas, J, “Ab Initio Powder X-Ray Diffraction Structural Analysis of Bispidine Based 1D Coordination Polymers: Insights into Their Guest Responsive Behaviour” *Dalton Transactions* ***48*** (44), (2019): 16756–16763.

[11]	Lippi, M.; Caputo, J.; Meneghetti, F.; Castellano, C.; Martí-Rujas, J.; Cametti, M, “Tuneable Solvent Adsorption and Exchange by 1D Bispidine-Based Mn (II) Coordination Polymers via Ligand Design” *Dalton Transactions* ***49*** (38), (2020): 13420–13429.

[43]	Bondi, A. van, “van der Waals Volumes and Radii” *The Journal of Physical Chemistry* **68**, (1964): 441–451.

[44]	Mesecke, K, “Hkl-Based Calculation of Total Scattering Patterns from Discrete and Low-Dimensional Structure Models Using TOPAS” *Applied Crystallography* ***58*** (1), (2025): 187-193.

[45]	Tran, Y. B. N.; Nguyen, P. T.; Luong, Q. T.; Nguyen, K. D. “Series of M-MOF-184 (M = Mg, Co, Ni, Zn, Cu, Fe) metal–organic frameworks for catalysis cycloaddition of CO_2_,” *Inorganic Chemistry* 59, (2020): 16747–16759.

[46]	Niwa, S.; Hashimoto, S.; Chen, D.; Toyao, T.; Shimizu, K. I.; Sadakiyo, M. “High ionic conduction in a robust anionic metal–organic framework containing Mg^2+^ under guest vapors,” *Dalton Transactions* 53, (2024): 12043–12046.

[47]	Li, G. A.; Deng, M.; Guo, W.; Yin, S.; Liu, Y. E.; Zhu, A. X.; Zaworotko, M. J. “Guest-induced gate-opening in a flexible MOF adsorbent that exhibits benzene/cyclohexane selectivity,” *Inorganic Chemistry Frontiers* 12, (2025): 3449–3455.

[48]	Tu, T. N.; Kwon, H. T.; Scheer, M.; Kim, J. “High toluene uptake at a trace concentration in a novel gallium-based metal–organic framework,” *Journal of Materials Chemistry A* 13, (2025): 9479–9485.

[49]	Hu, P.; Liang, X.; Yaseen, M.; Sun, X.; Tong, Z.; Zhao, Z.; Zhao, Z. “Preparation of highly hydrophobic novel N-coordinated UiO-66(Zr) with dopamine via fast mechanochemical method for (CHO-/Cl-)-VOCs competitive adsorption in humid environment,” *Chemical Engineering Journal* 332, (2018): 608–618.

[50]. Bullot, L.; Vieira-Sellaï, L.; Chaplais, G.; Simon-Masseron, A.; Daou, T. J.; Patarin, J.; Fiani, E. “Adsorption of 1,2-dichlorobenzene and 1,2,4-trichlorobenzene in nano- and microsized crystals of MIL-101(Cr): static and dynamic gravimetric studies,” *Environmental Science and Pollution Research* 24, (2017): 26562–26573.

[51]	Wang, S.-Q.; Darwish, S.; Xu, S.; Wang, R.; Zaworotko, M. J. “Aromatic guest-induced layer expansion and phase switching in a 2D square lattice coordination network,” *Dalton Transactions* 55, (2026): 8033–8039.
